# The gut microbiota regulates autism-like behavior by mediating vitamin B_6_ homeostasis in EphB6-deficient mice

**DOI:** 10.1186/s40168-020-00884-z

**Published:** 2020-08-20

**Authors:** Ying Li, Zheng-Yi Luo, Yu-Ying Hu, Yue-Wei Bi, Jian-Ming Yang, Wen-Jun Zou, Yun-Long Song, Shi Li, Tong Shen, Shu-Ji Li, Lang Huang, Ai-Jun Zhou, Tian-Ming Gao, Jian-Ming Li

**Affiliations:** 1 Department of Pathology, Sun Yat-Sen Memorial Hospital, Sun Yat-Sen University, Guangzhou, 510120 People’s Republic of China; 2 State Key Laboratory of Organ Failure Research, Key Laboratory of Mental Health of the Ministry of Education, Guangdong-Hong Kong-Macao Greater Bay Area Center for Brain Science and Brain-Inspired Intelligence, Guangdong Province Key Laboratory of Psychiatric Disorders Collaborative Innovation Center for Brain Science, Department of Neurobiology, School of Basic Medical Sciences, Southern Medical University, Guangzhou, 510515 People’s Republic of China; 3 Department of Pathology, Soochow University Medical School, Suzhou, 215123 People’s Republic of China

**Keywords:** Gut microbiota, ASD, EphB6, Vitamin B_6_, Dopamine, E/I balance

## Abstract

**Background:**

Autism spectrum disorder (ASD) is a developmental disorder, and the effective pharmacological treatments for the core autistic symptoms are currently limited. Increasing evidence, particularly that from clinical studies on ASD patients, suggests a functional link between the gut microbiota and the development of ASD. However, the mechanisms linking the gut microbiota with brain dysfunctions (gut-brain axis) in ASD have not yet been full elucidated. Due to its genetic mutations and downregulated expression in patients with ASD, *EPHB6*, which also plays important roles in gut homeostasis, is generally considered a candidate gene for ASD. Nonetheless, the role and mechanism of *EPHB6* in regulating the gut microbiota and the development of ASD are unclear.

**Results:**

Here, we found that the deletion of EphB6 induced autism-like behavior and disturbed the gut microbiota in mice. More importantly, transplantation of the fecal microbiota from EphB6-deficient mice resulted in autism-like behavior in antibiotic-treated C57BL/6J mice, and transplantation of the fecal microbiota from wild-type mice ameliorated the autism-like behavior in EphB6-deficient mice. At the metabolic level, the disturbed gut microbiota in EphB6-deficient mice led to vitamin B_6_ and dopamine defects. At the cellular level, the excitation/inhibition (E/I) balance in the medial prefrontal cortex was regulated by gut microbiota-mediated vitamin B_6_ in EphB6-deficient mice.

**Conclusions:**

Our study uncovers a key role for the gut microbiota in the regulation of autism-like social behavior by vitamin B_6_, dopamine, and the E/I balance in EphB6-deficient mice, and these findings suggest new strategies for understanding and treating ASD.

Video abstract.

## Background

Autism spectrum disorder (ASD), which affects approximately 1% of the population around the world, is mainly characterized by impaired social interaction and communication and restricted and repetitive behavior [1]. Although early behavioral and educational interferences have shown effective ameliorative roles on autistic symptoms of ASD patients, the effective pharmacological therapies for the treatment of core autistic symptoms remain limited [1, 2].

Accumulating evidence shows that the gut-brain-microbiota axis plays a key role in regulating homeostasis of the human body. Gut microorganisms reportedly participate in many neuropsychiatric disorders, such as anxiety disorders, depression [3], and epilepsy [4]. In most ASD patients, changes in gut microorganisms and serious gastrointestinal problems have been observed [5–7]. Interestingly, several studies have found that the gut microbiota play important role in modulating the ASD-like phenotypes of mice [8–10]. A clinical study showed that microbiota transfer therapy can improve gastrointestinal problems and autistic symptoms in ASD patients aged 7 to 16 years, and this benefit can last for 2 years [11, 12]. These studies suggest that the gut-brain-microbiota axis might have a significant impact on the development of ASD. However, the contribution of the gut microbiota to the dysregulation of brain function has not been fully elucidated.

*EPHB6*, which belongs to the Eph family of receptor tyrosine kinases, is located on chromosome 7q. In 1998, two-stage genome research on susceptibility loci in autism found transcripts mapped to the chromosome 7q region that are associated with a predisposition to autism, including *EPHB6* [13]. More recent studies have suggested that *EPHB6* is a candidate ASD-associated gene [14–16], and recent genomic studies have found that *EPHB6* is mutated in some ASD patients [17, 18]. Most importantly, transcriptome analyses have shown that *EPHB6* is downregulated in ASD patients [19, 20]. Although *EPHB6* plays an important role in regulating Eph receptor signaling networks, T cell functions, development of intestinal epithelium, and epithelial homeostasis [21–23], the role and mechanisms of *EPHB6* involved in regulation of the gut microbiota and ASD remain unclear.

In our study, we found that EphB6 is functionally associated with ASD and regulates autism-like social behavior by gut microbiota-mediated vitamin B_6_ and dopamine. More importantly, we established the functional link between dysregulated gut microbiota and excitation/inhibition (E/I) imbalance in the medial prefrontal cortex (mPFC), a key gut-brain functional axis, in EphB6-deficient mice.

## Results

### The deletion of EphB6 led to autism-like behavior and gut microbial disturbance in mice

Although *EPHB6* has been identified as a candidate gene associated with ASD, whether and how *EPHB6* functions in ASD remain unclear. To address these unanswered questions, we established EphB6-knockout (KO) mice and found that EphB6 was deleted in different tissues, including the colon, brain, lung, and spleen, in these mice compared with EphB6^+/+^ (wild-type, WT) mice (Additional file 1: Figure S1c-d). However, the brain and body weights, the body length, and the daily dietary consumption were similar between the two groups of mice despite the deletion of EphB6 (Additional file 1: Figure S1e-h).

**Fig. 1:**
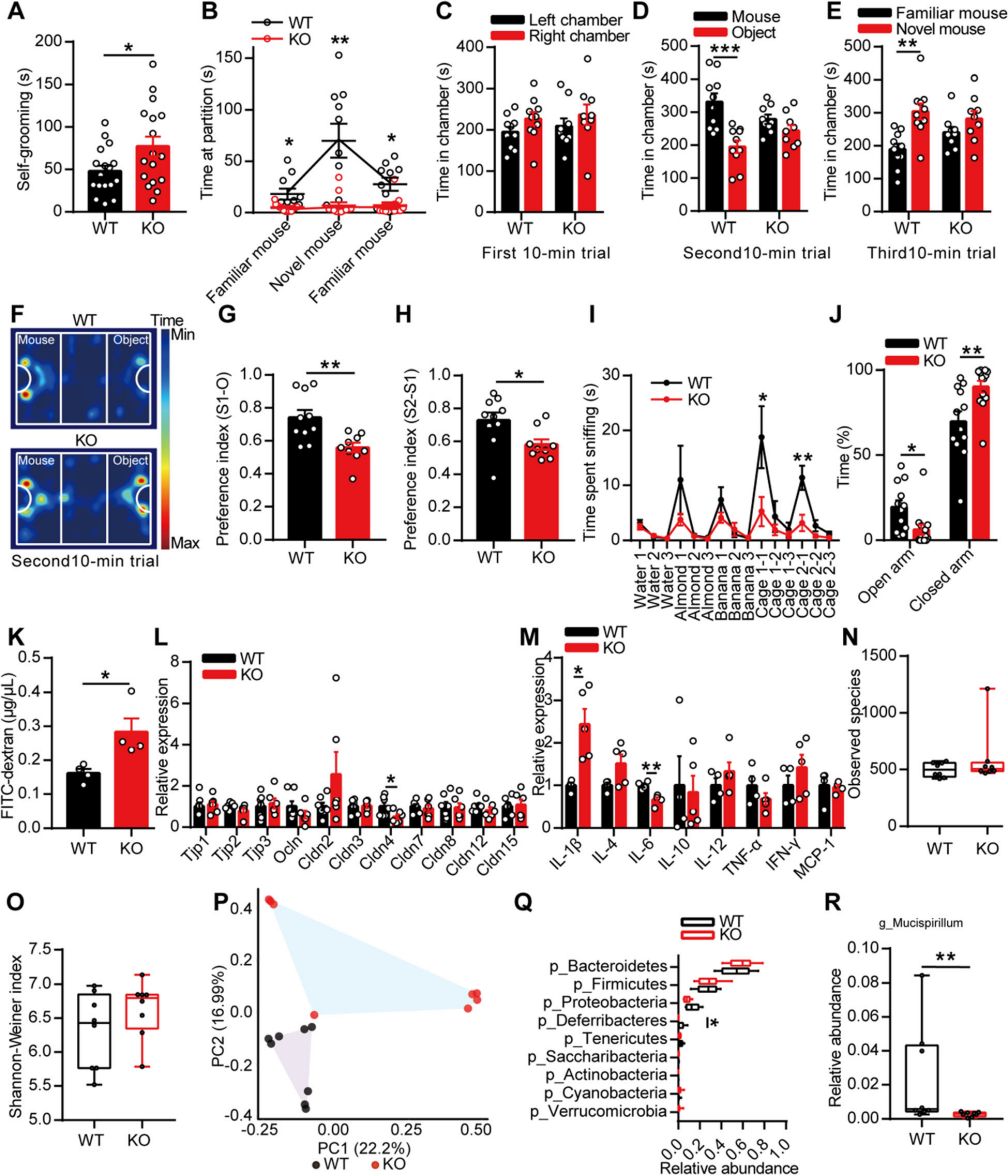
The deletion of EphB6 led to autism-like behavior and gut microbial disturbance in mice. **a** The **8**-week-old male KO mice spent more time self-grooming than WT mice. *n* = 17 mice for each group. **b** In social partition test, KO mice spent less time sniffing the partition than WT mice. *n* = 10, 11 mice respectively. **c**–**h** In three-chambered social approach task, time spent in chambers during different 10-min trials (**c**–**e**), trajectory diagram during the second 10-min trial (**f**) were showed. KO mice showed less preference for the social mouse over the object (**g**) and less preference for the novel social mouse over the familiar social mouse (**h**) than WT mice. *n* = 10, 9 mice respectively. **i** In olfactory habituation/dishabituation test, KO mice spent less time sniffing social odors than WT mice. *n* = 11 mice for each group. **j** In elevated plus maze test, KO mice spent less time in open arm and more time in closed arm than WT mice. *n* = 12, 13 mice respectively. **k** The intestinal permeability of 8-week-old WT and KO mice was detected using FITC-dextran. *n* = 4 mice for each group. **l** The mRNA expressions of tight junction molecules were detected in colon of 8-week-old WT and KO mice. *n* = 7, 6 mice respectively. **m** The mRNA expressions of cytokines were detected in colon of 8-week-old WT and KO mice. *n* = 4, 5 mice respectively. **n**–**r** 16S rRNA gene sequencing of gut microbiota of 8-week-old WT and KO mice. The species richness (**n**) and diversity (**o**) of gut microbiota were similar, while the microbial composition (**p**) was different between the two groups. Relative abundance of different bacteria in phylum level was showed (**q**). At genus level, the relative abundance of *Mucispirillumn* was decreased in KO mice (**r**). *n* = 8 mice for each group. Data shown are mean ± SEM or median ± IQR. Two-tailed unpaired student’s *t* test (**a**, **c**–**e**, **g**–**h**, **j**–**m**), Mann-Whitney test (**n**–**o**, **q**, **r**), mixed design ANOVA with genotype as independent factor and stimuli/trials as repeated-measure factor (**b**, **i**), anosim analysis (**p**). *p < 0.05; **p < 0.01; ***p < 0.001. *WT* EphB6^+/+^ mice, *KO* EphB6^−/−^ mice, *FITC* fluorescein isothiocyanate. Statistical values are presented in Additional file 3: Table S2

Patients with ASD often display repetitive stereotyped behavior and social deficits. Interestingly, we found that the KO mice spent more time self-grooming than the WT mice (Fig. 1a). In the marble burying test, the KO mice buried similar marbles as the WT mice (Additional file 1: Figure S1j), and in the social partition test, the KO mice spent less time sniffing at the partition, regardless of whether a familiar or novel mouse was placed in the cage, than the WT mice (Fig. 1b). In the three-chambered social approach task, both the WT and KO mice spent similar lengths of time in bilateral chambers during the first 10-min trial, which indicated that the experimental environment was normal (Fig. 1c). However, the KO mice spent a similar length of time in chambers with an unfamiliar mouse or inanimate object (Fig. 1d) and also showed less preference for the social mouse (stranger 1) over the object than the WT mice (Fig. 1f–g). If a novel social partner (stranger 2) was placed in the empty wire cage, the KO mice still spent a similar length of time in the two chambers (Fig. 1e) and showed less preference for the novel mouse over the familiar mouse than the WT mice (Fig. 1h). These results sufficiently confirmed that the KO mice exhibited abnormal social interaction. Olfactory cues have generally been considered to be of the utmost importance in communication among mice [24, 25]. In the olfactory habituation/dishabituation test, repeated presentation of cotton swabs saturated with the same odor resulted in increasingly decreased lengths of time spent sniffing at cotton swabs, and the presentation of cotton swabs saturated with a new odor increased the time spent sniffing; these findings were obtained with both the WT and KO mice. However, the KO mice showed less interest in cotton swabs saturated with social odor than the WT mice (Fig. 1i). These results indicated that the KO mice exhibited communication deficits, even though their ability to discriminate and habituate different odors was normal.

ASD is often accompanied by other mental diseases, such as hyperactivity, anxiety, and intellectual disability. In the open field test, the KO mice showed the same locomotor activities and spent almost the same time in the center area as the WT mice (Additional file 1: Figure S1k-l). In the elevated-plus-maze test, the KO mice spent less time in the open arm and more time in the closed arm than the WT mice (Fig. 1j), which implied that the KO mice displayed anxiety-like behavior. In the Morris water maze, the KO mice exhibited normal spatial learning and memory, similarly to the WT mice (Additional file 1: Figure S1m-o). Collectively, the results showed that the deletion of EphB6 in mice resulted in autism-like behavior, including stereotyped behavior and social deficits, accompanied by anxiety-like behavior, but did not result in any evidence of intellectual disability.

Eph/ephrin signaling reportedly modulates gut epithelial development and homeostasis, and it is also generally accepted that many ASD patients present gastrointestinal (GI) symptoms [5, 6, 26] and a changed gut microbiota composition [7]. We then questioned whether KO mice would suffer from GI problems. Measurement of the intestinal permeability by fluorescein isothiocyanate (FITC)-dextran revealed that the intestinal permeability of KO mice was significantly increased compared with that of the WT mice (Fig. 1k). Accordingly, the mRNA expression of Cldn4, a tight junction molecule, in the colon of KO mice was lower than that in the colon of WT mice (Fig. 1l). In addition, we found that the colon of the KO mice presented substantially increased mRNA expression of IL-1β, a proinflammatory factor, and decreased expression of IL-6, which exerts an anti-inflammatory effect, compared with that of the WT mice (Fig. 1m). The GI problems in the KO mice were not accompanied by morphological changes in the distal ileum, proximal colon, liver, or lung (Additional file 1: Figure S1p).

The integrity of the intestinal mucosa is important for maintaining the balance of the ecological environment in the animals’ gut. We then detected the fecal microbial populations of mice by 16S rRNA gene sequencing. No differences in the microbial species richness and diversity were found between the two groups (Fig. 1n, o). Notably, a principal coordinates analysis of the Bray-Curtis distance showed that the fecal microbiota of the KO mice clustered differently from that of the WT mice (Fig. 1p), which indicated that the gut microbial composition differed between the two groups. At the phylum level, the differences between the two groups were caused by a decreased abundance of *Deferribacteres* in the fecal microbiota of the KO mice (Fig. 1q). At the genus level, *Mucispirillum*, which is a genus belonging to the phylum *Deferribacteres*, was decreased in the fecal microbiota of the KO mice (Fig. 1r). In general, our results indicated that the deletion of EphB6 in mice resulted in increased intestinal permeability and changes in the gut microbial composition.

Many studies have indicated that GI problems and the behavioral abnormalities associated with ASD always appear in parallel in patients [5]. We thus questioned which of these symptoms appears first in the KO mice and found that the microbial species richness and diversity did not differ between the 3/4-week-old WT and KO mice (Additional file 1: Figure S2a-b). The principal coordinates analysis revealed that the gut microbiota of 4-week-old KO mice clustered differently from that of 4-week-old WT mice (Additional file 1: Figure S2d), whereas the gut microbiota of 3-week-old KO mice clustered similarly to that of 3-week-old WT mice (Additional file 1: Figure S2c). In addition, 4-week-old, but not 3-week-old, KO mice showed increased self-grooming and decreased interest in social odors compared with same-aged WT mice (Additional file 1: Figure S2e-h). These results further implied a possible relationship between the abnormal behavior and gut microbial dysbiosis in mice with deletion of EphB6.

### Transplantation of the fecal microbiota from EphB6-deficient mice caused autism-like behavior in SPF C57BL/6J mice

**Fig. 2:**
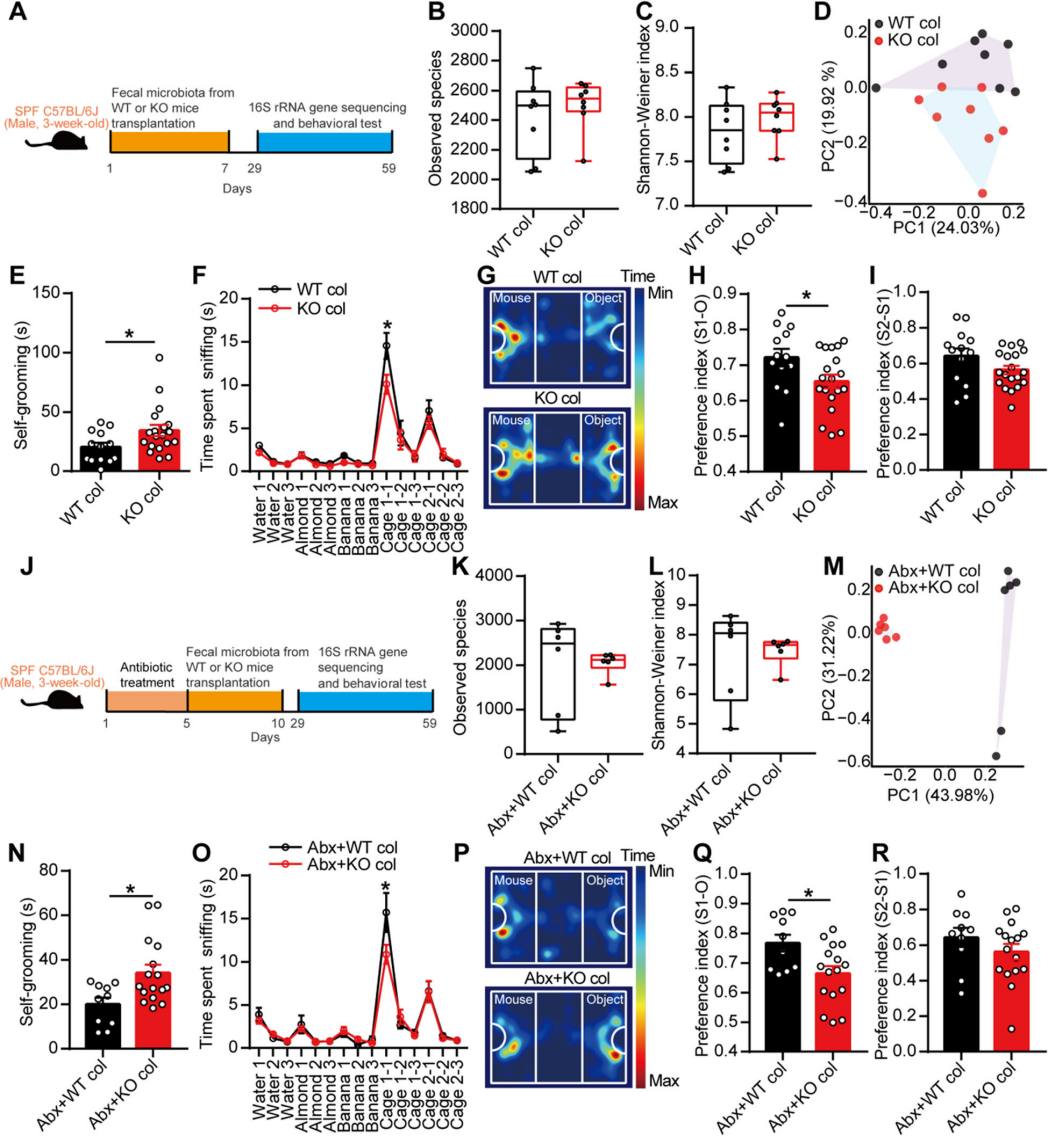
Transplantation of the fecal microbiota from EphB6-deficient mice caused autism-like behavior in 3-week-old SPF C57BL/6J mice. **a**–**i** Schematic of the fecal microbiota transplantation (**a**). The 3-week-old SPF male C57BL/6J mice were orally gavaged with the fecal microbiota from 8-week-old male WT or KO mice (each contained eight healthy mice from at least three cages) for 1 week. After 3 weeks, the fecal microbiota of the treated C57BL/6J mice were sequenced (**b**–**d**, eight treated C57BL/6J mice of each group were selected randomly from at least three cages) and self-grooming test (**e**), olfactory habituation/dishabituation test (**f**), three-chambered social approach task (**g**–**i**), open field test, and elevated plus maze test were conducted with an interval of at least 2 days (**e**–**i**, *n* = 13, 19 mice respectively). **j**–**r** Schematic of the fecal microbiota transplantation (**j**). The 3-week-old SPF male C57BL/6J mice were orally gavaged with antibiotics (ampicillin, vancomycin, neomycin, metronidazole) for 5 days and then orally gavaged with the fecal microbiota from 8-week-old male WT or KO mice (each contained eight healthy mice from at least three cages) for another 5 days. After 19 days, the fecal microbiota of the treated C57BL/6J mice were sequenced (**k**–**m**, six treated C57BL/6J mice of each group were selected randomly from at least two cages) and self-grooming test (**n**), olfactory habituation/dishabituation test (**o**), three-chambered social approach task (**p**–**r**), open field test, and elevated plus maze test were conducted with an interval of at least 2 days (**n**–**r**, *n* = 10, 16 mice respectively). Data shown are mean ± SEM or median ± IQR. Two-tailed unpaired student’s *t* test (**e**, **h**, **i**, **n**, **q**, **r**), Mann-Whitney test (**b**, **c**, **k**, **l**), mixed design ANOVA with genotype as independent factor and stimuli/trials as repeated-measure factor (**f**, **o**), anosim analysis (**d**, **m**). *p < 0.05. *WT col* or *KO col* colonized with the fecal microbiota from EphB6^+/+^ or EphB6^−/−^ mice, *Abx* pretreated with antibiotics (ampicillin, vancomycin, neomycin, metronidazole). Statistical values are presented in Additional file 3: Table S2

ASD is generally considered a neurodevelopmental disorder; postnatal developmental disorder can also cause autism in patients [27], and the postnatal mutation of Nrxn1 in neurons leads to autism-like behavior in mice [28]. Additionally, the gut microbiota of ASD patients could induce autism-like behavior in mice [7]. Therefore, to study the relationship between gut microbial dysbiosis and autism-like behavior in mice with deletion of EphB6, we gavaged the fecal microbiota from 8-week-old male WT or KO mice to 3-week-old SPF male C57BL/6J mice for 1 week (Fig. 2a). Three weeks after the gavage of fecal microbiota, the gut microbial composition of SPF C57BL/6J mice treated with the fecal microbiota from the KO mice differed from that of SPF C57BL/6J mice treated with the fecal microbiota from the WT mice (Fig. 2b–d). More interestingly, C57BL/6J mice that were gastrically perfused with the fecal microbiota from the KO mice displayed increased self-grooming (Fig. 2e) and partially decreased social behavior (Fig. 2f–i) compared with the control mice. The two groups of mice showed similar behaviors in the open field test and elevated plus maze test (Additional file 1: Figure S3a-d). Furthermore, we orally gavaged the suspending solution of fecal microbiota from the WT or KO mice to antibiotic-pretreated SPF male C57BL/6J mice. After pretreatment with antibiotics for 5 days, 3-week-old SPF male C57BL/6J mice was gavaged orally with the fecal microbiota of 8-week-old male WT or KO mice for 5 days (Fig. 2j). Approximately 2 weeks after fecal microbial colonization, we similarly found that the gut microbiota of SPF C57BL/6J mice treated with the fecal microbiota from the KO mice clustered differently from that of the control mice (Fig. 2k–m). We subsequently found that C57BL/6J mice that were gastrically perfused with the fecal bacteria from the KO mice showed increased self-grooming (Fig. 2n) and partially decreased social behavior (Fig. 2o–r). Additionally, the two groups of mice showed similar behaviors in the open field test and elevated-plus-maze test (Additional file 1: Figure S3e-h). Moreover, the fecal microbiota from 4-week-old, but not 3-week-old, KO mice induced increased self-grooming and partial social deficits in 3-week-old SPF C57BL/6J mice compared with C57BL/6J mice gavaged with fecal microbiota from same-aged WT mice (Additional file 1: Figure S4a-h). Collectively, the fecal microbiota from EphB6-deficient mice caused increased self-grooming and partially impaired social behavior in C57BL/6J mice.

**Fig. 3:**
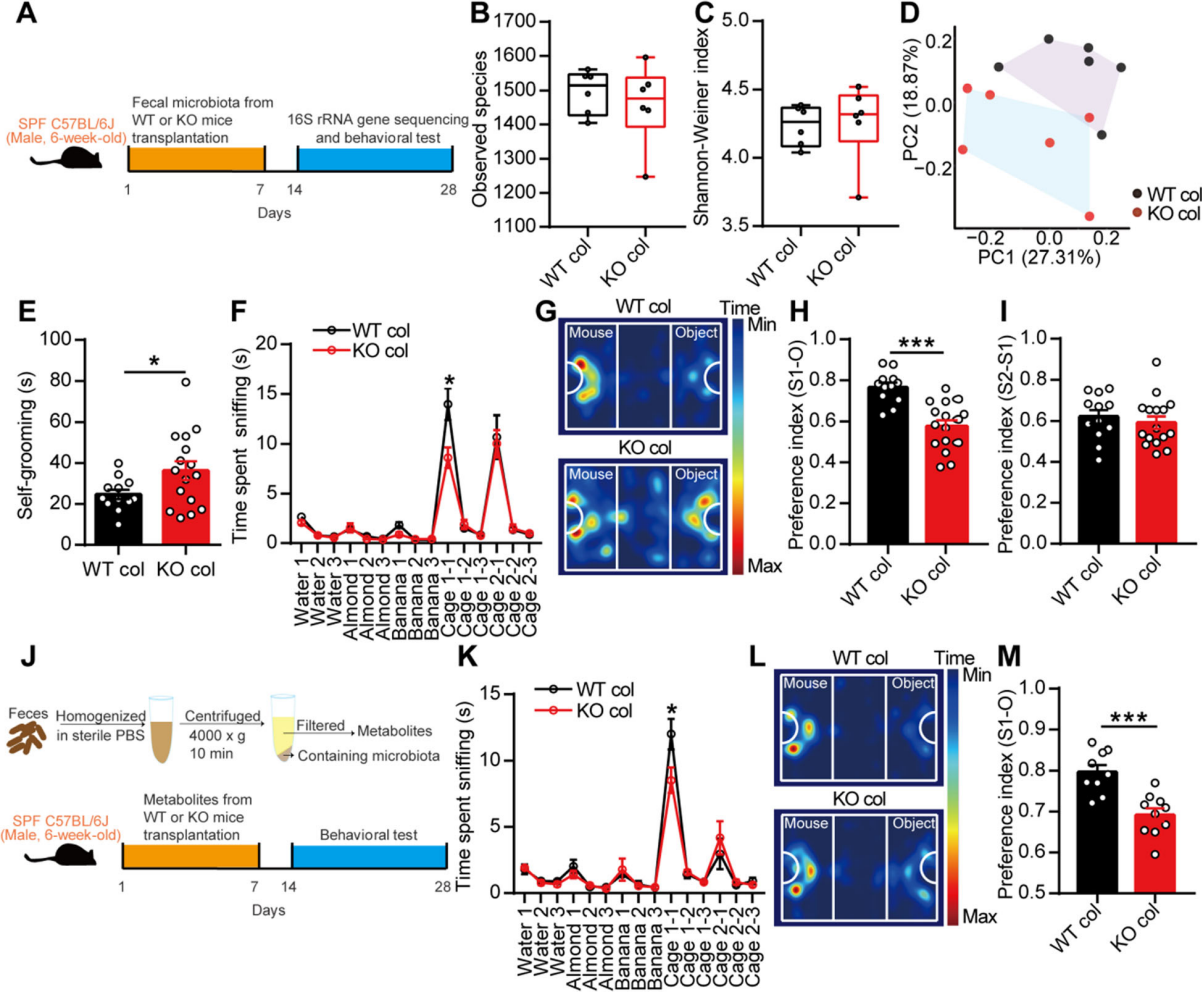
Fecal microbiota transplantation from EphB6-deficient mice partially induced social deficits in 6-week-old SPF C57BL/6J mice. **a**–**i** Schematic of the fecal microbiota transplantation (**a**). The 6-week-old SPF male C57BL/6J mice were orally gavaged with fecal microbiota from 8-week-old male WT or KO mice for 1 week. After 1 week, the fecal microbiota of the treated C57BL/6J mice were sequenced (**b**–**d**, *n* = 6 mice for each group) and self-grooming test (**e**), olfactory habituation/dishabituation test (**f**), three-chambered social approach test (**g**–**i**) were conducted with an interval of at least 2 days (**e**–**i**, *n* = 12, 16 mice respectively). **j**–**m** Fecal metabolites from 8-week-old male WT and KO mice were orally gavaged to 6-week-old SPF male C57BL/6J mice for 1 week (**j**). After 1 week, olfactory habituation/dishabituation test (**k**) and three-chambered social approach task (**l**, **m**) were conducted with an interval of at least 2 days. *n* = 9, 10 mice respectively. Data shown are mean ± SEM or median ± IQR. Two-tailed unpaired student’s *t* test (**e**, **h**, **i**, **m**), Mann-Whitney test (**b**, **c**), mixed design ANOVA with genotype as independent factor and stimuli/trials as repeated-measure factor (**f**, **k**), anosim analysis (**d**). *p < 0.05; ***p < 0.001. WT col or KO col, colonized with fecal microbiota or fecal metabolites from EphB6^+/+^ mice or EphB6^−/−^ mice. Statistical values are presented in Additional file 3: Table S2

We subsequently questioned whether the gut microbiota continue to play a role in autism-like behavior in adult mice. First, we orally gavaged a mixture of antibiotics to 6-week-old male SPF C57BL/6J mice for 1 week and found that this antibiotic treatment greatly disrupted the gut microbiota and induced decreased self-grooming and partial social deficits in young adult C57BL/6J mice (Additional file 1: Figure S5a-i). These results indicated that the gut microbiota was related to autism-like behavior even in adult mice and that different gut microbiota compositions likely contributed to different behaviors, such as self-grooming and social behavior. We then gavaged the fecal microbiota from 8-week-old male WT or KO mice directly to 6-week-old SPF male C57BL/6J mice for 1 week and found that the fecal microbiota from KO mice also induced a disturbed gut microbiota, increased self-grooming, and partial social deficits in adult C57BL/6J mice (Fig. 3a–i). Unexpectedly, we also found that metabolites of the gut microbiota from the KO mice induced partial social deficits in C57BL/6J mice (Fig. 3j–m). The gut microbiota without metabolites from the KO mice still caused partial social deficits in C57BL/6J mice (Additional file 1: Figure S5j-m).

Overall, our results indicated that the gut microbiota plays an important role in autism-like behavior, even in adult mice.

### Transplantation of the fecal microbiota from wild-type mice ameliorated autism-like behavior in adult EphB6-deficient mice

**Fig. 4:**
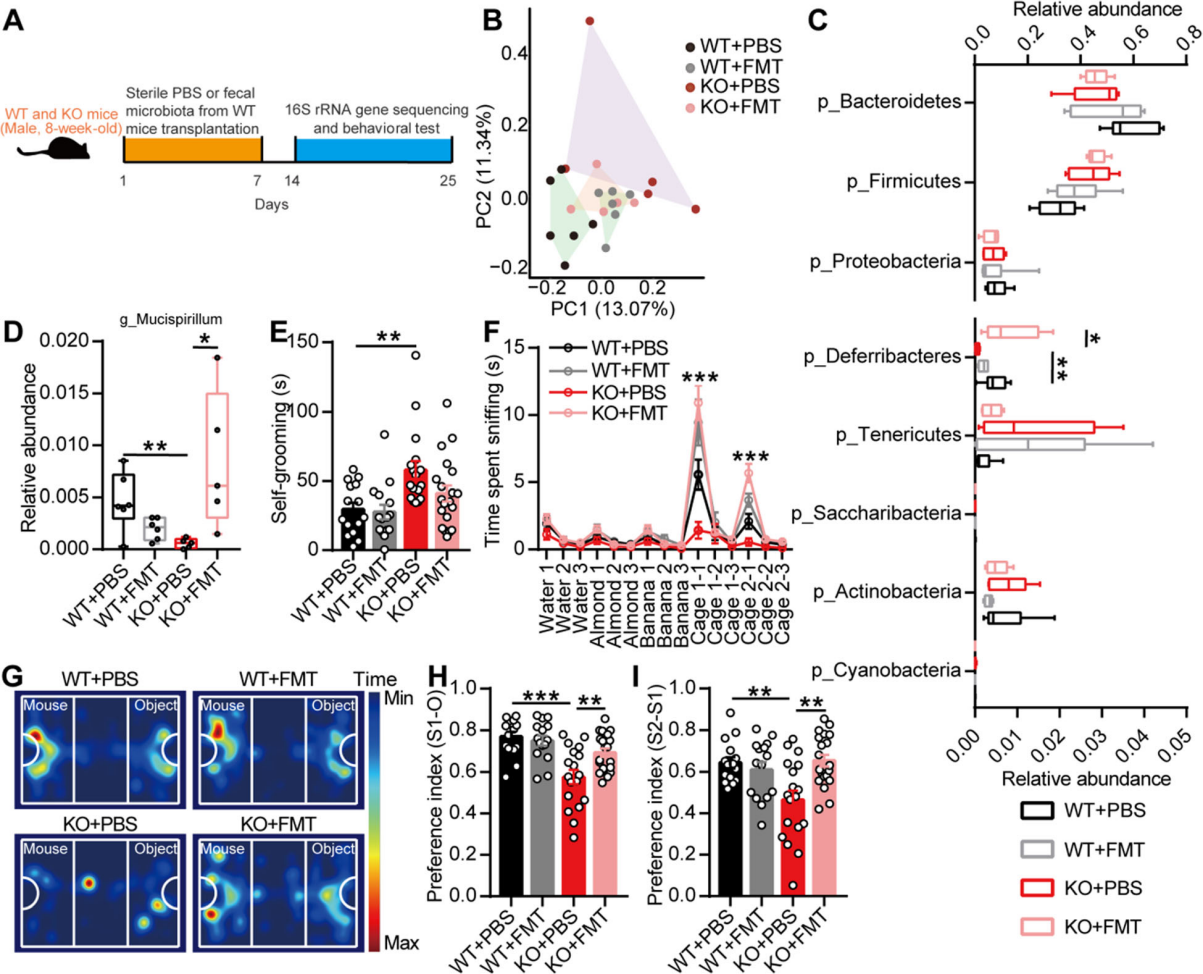
Transplantation of the fecal microbiota from wild-type mice ameliorated autism-like behavior in adult EphB6-deficient mice. **a** Schematic of the the fecal microbiota transplantation. The 8-week-old male WT and KO mice were orally gavaged with the fecal microbiota from 8-week-old male WT mice (eight healthy mice from at least three cages) or sterile PBS for 1 week. After 1 week, the fecal microbiota of the treated WT and KO mice were sequenced (**b**–**d**) and behavioral tests were conducted with an interval of at least 2 days (**e**–**i**). **b**–**d** 16S rRNA gene sequencing of the fecal microbiota from mice. Principal coordinates analysis of Bray-Curtis distance (**b**), the relative abundance of *Deferribacteres* (**c**) at phylum level, and the relative abundance of *Mucispirillum* (**d**) at genus level were showed. At phylum level, the range of 0–0.8 on *x* axis was used for the relative abundance of p_Bacteroidetes, p_Firmicutes, and p_Proteobacteria and the range of 0–0.05 on *x* axis was used for other bacteria. *n* = 6, 6, 5, 5 mice respectively. **e**–**i** Self-grooming test (**e**), olfactory habituation/dishabituation test (**f**), and three-chambered social approach task (**g**–**i**) were performed. *n* = 15, 15, 18, 20 mice respectively. Data shown are mean ± SEM or median ± IQR. One-way ANOVA (**e**, **h**, **i**), Kruskal-Wallis test (**c**, **d**), mixed design ANOVA with genotype as independent factor and stimuli/trials as repeated-measure factor (**f**), anosim analysis (**b**). *p < 0.05; **p < 0.01; ***p < 0.001. *WT* EphB6^+/+^ mice, *KO* EphB6^−/−^ mice, *FMT* fecal microbiota transplantation, *PBS* phosphate-buffered saline. Statistical values are presented in Additional file 3: Table S2

No previous study has focused on the effectiveness of microbiota transplantation in adult ASD patients. We subsequently orally gavaged the fecal microbiota from 8-week-old male WT mice to 8-week-old KO mice for 1 week. A week later, we found that the gut microbiota of the KO mice gavaged with the fecal microbiota of the WT mice clustered differently from that of the KO mice gavaged with sterile PBS (Fig. 4b). The phylum-level analysis revealed that the relative abundance of *Deferribacteres* was increased in the KO mice gavaged with the fecal microbiota of the WT mice (Fig. 4c). At the genus level, *Mucispirillum*, which is a genus belonging to the phylum *Deferribacteres*, was also increased in the KO mice treated with the fecal microbiota of the WT mice (Fig. 4d).

A functional analysis revealed that the KO mice exhibited increased social behavior (Fig. 4f–i) after gavage with the fecal microbiota from the WT mice, and a decreased tendency of self-grooming (Fig. 4e). These results indicated that gut microbial dysbiosis was responsible for autism-like behavior in mice with deletion of EphB6.

### Gut microbiota-mediated vitamin B_6_ homeostasis regulated social behavior in EphB6-deficient mice

Because the abnormal behaviors were likely due to brain-related problems, we attempted to determine how the gut microbiota affected the brain and subsequently caused autism-like behavior in EphB6-deficient mice.

First, we attempted to identify the key region of the brain affected by the dysregulated gut microbiota in mice with deletion of EphB6 and that was responsible for the resulting autism-like behavior. Studies on ASD patients or mouse models have shown that the hippocampus, cerebellum, and mPFC are implicated in ASD [29, 30]. After processed with a three-chambered social approach task, the protein expression of c-Fos in the mPFC of the KO mice was significantly higher than that in the mPFC of the WT mice (Additional file 1: Figure S6a-c). ASD is generally considered to be caused by an increased ratio of synaptic excitation and inhibition, and ASD children exhibit elevations in the resting-state neuronal activity [31]. Therefore, whether the mPFC is modulated by the gut microbiota of the KO mice needs to be further investigated. Because the mPFC tissue was too small for some experiments, we used PFC tissue from mice in our subsequent study.

The first question we asked was whether the bacteria could directly modulate the mPFC. Undoubtedly, we did not detect any bacterial DNA or colonies in the PFC tissues of the WT or KO mice (Additional file 1: Figure S6d-e). Because metabolites of the gut microbiota from the KO mice also induced social deficits in C57BL/6J mice, we hypothesized that some substances that had been affected by gut microbial dysbiosis caused social deficits in the KO mice.

**Fig. 5:**
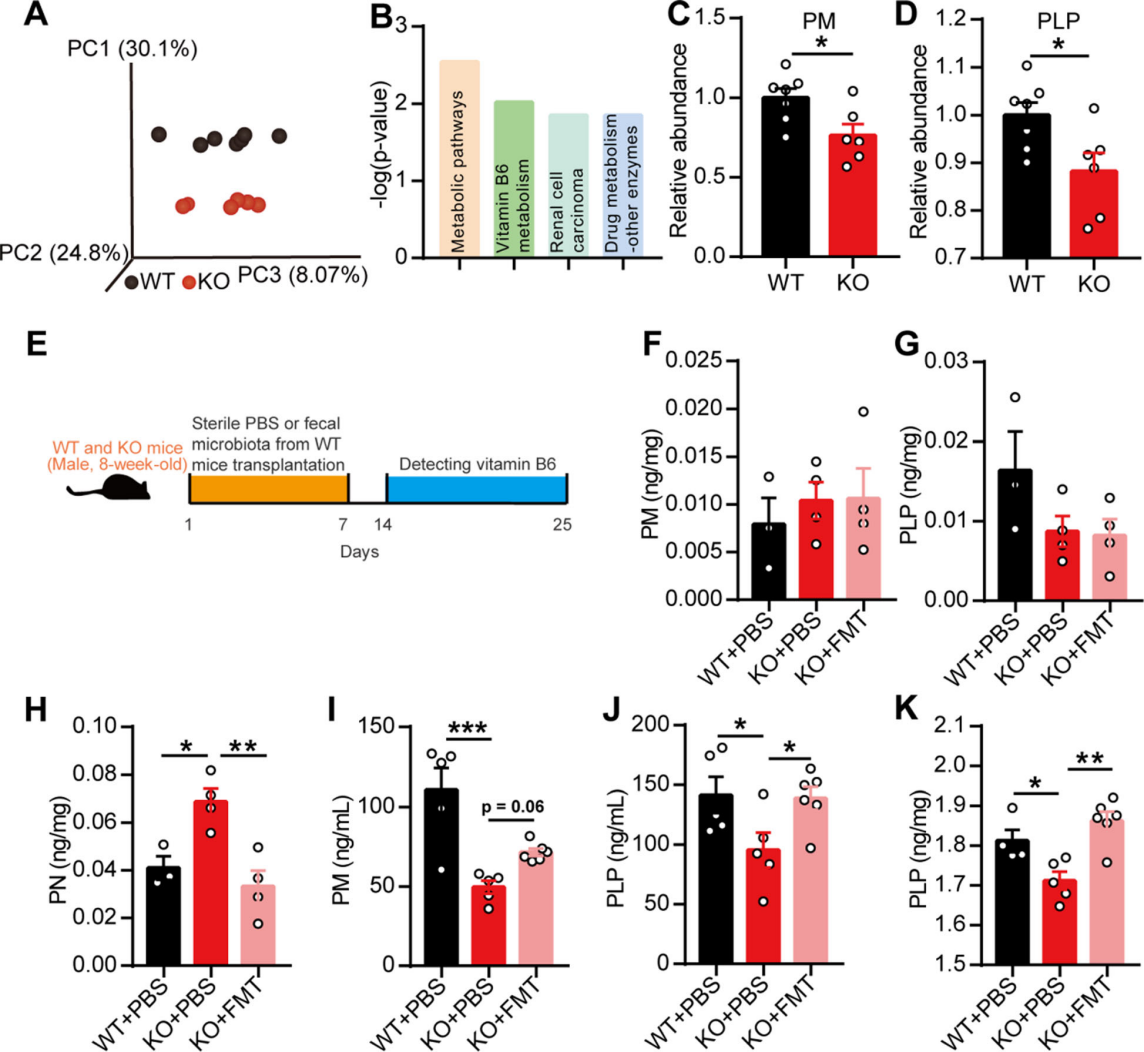
Gut microbiota regulated vitamin B_6_ in EphB6-deficient mice. **a**–**d** In non-targeted metabolomics analysis, the metabolites in PFC of 8-week-old male WT and KO mice were differently clustered by orthogonal partial least squares discriminant analysis (**a**). The enriched KEGG pathways associated with differential metabolites (**b**), the relative abundance of pyridoxamine (PM, **c**), and pyridoxal 5′-phosphate (PLP, **d**) were showed. *n* = 7, 6 mice respectively. **e**–**k** The fecal microbiota from 8-week-old WT mice or PBS were gavaged to 8-week-old WT or KO mice for 1 week (**e**). One week later, the level of PM (**f**), PLP (**g**), and pyridoxine (PN, **h**) in feces of mice were detected. *n* = 3, 4, 4 mice respectively. The level of PM and PLP in plasma (**i**, **j**, *n* = 5, 5, 6 mice respectively) and level of PLP in PFC (**k**, *n* = 4, 5, 6 mice respectively) of mice were also detected. Data shown are mean ± SEM. R (**b**–**d**), one-way ANOVA (**f**–**k**). *p < 0.05; **p < 0.01; ***p < 0.001. WT, EphB6^+/+^ mice; KO, EphB6^−/−^ mice; *FMT* fecal microbiota transplantation, *PBS* phosphate-buffered saline, *PFC* prefrontal cortex, *PM* pyridoxamine, *PLP* pyridoxal 5′-phosphate, *PN* pyridoxine. Statistical values are presented in Additional file 3: Table S2

To identify the significantly changed metabolites, we detected the metabolites in the target tissue, that is, the PFC of the KO mice, using nontargeted metabolomics strategies. Surprisingly, the metabolites in the PFC showed significant differences between the two groups of mice, as demonstrated by orthogonal partial least squares discriminant analysis (Fig. 5a). A KEGG pathway analysis identified four pathways that were significantly enriched in the differentially changed metabolites, and these included the vitamin B_6_ metabolism pathway due to the decreased relative abundances of pyridoxamine (PM) and pyridoxal 5′-phosphate (PLP) in the PFC of the KO mice (Fig. 5b–d).

Vitamin B_6_ in the body is mainly derived from diet and gut bacteria synthesis and is then absorbed in the intestine. We then detected the levels of vitamin B_6_ in the feces, blood, and PFC of mice and found that the EphB6-deficient mice presented increased fecal levels of pyridoxine (PN), decreased plasma levels of PM and PLP, and decreased levels of PLP in the PFC (Fig. 5e–k). One week after gavage, the KO mice gavaged with the fecal microbiota from the WT mice exhibited decreased levels of PN in feces and tended to show increased levels of PM in plasma and increased levels of PLP in plasma and the PFC compared with the KO mice gavaged with sterile PBS (Fig. 5e–k). These results indicated that the gut microbiota regulated the level of vitamin B_6_ in the feces, blood, and PFC of mice, probably by regulating the absorption of vitamin B_6_ in intestine.

**Fig. 6:**
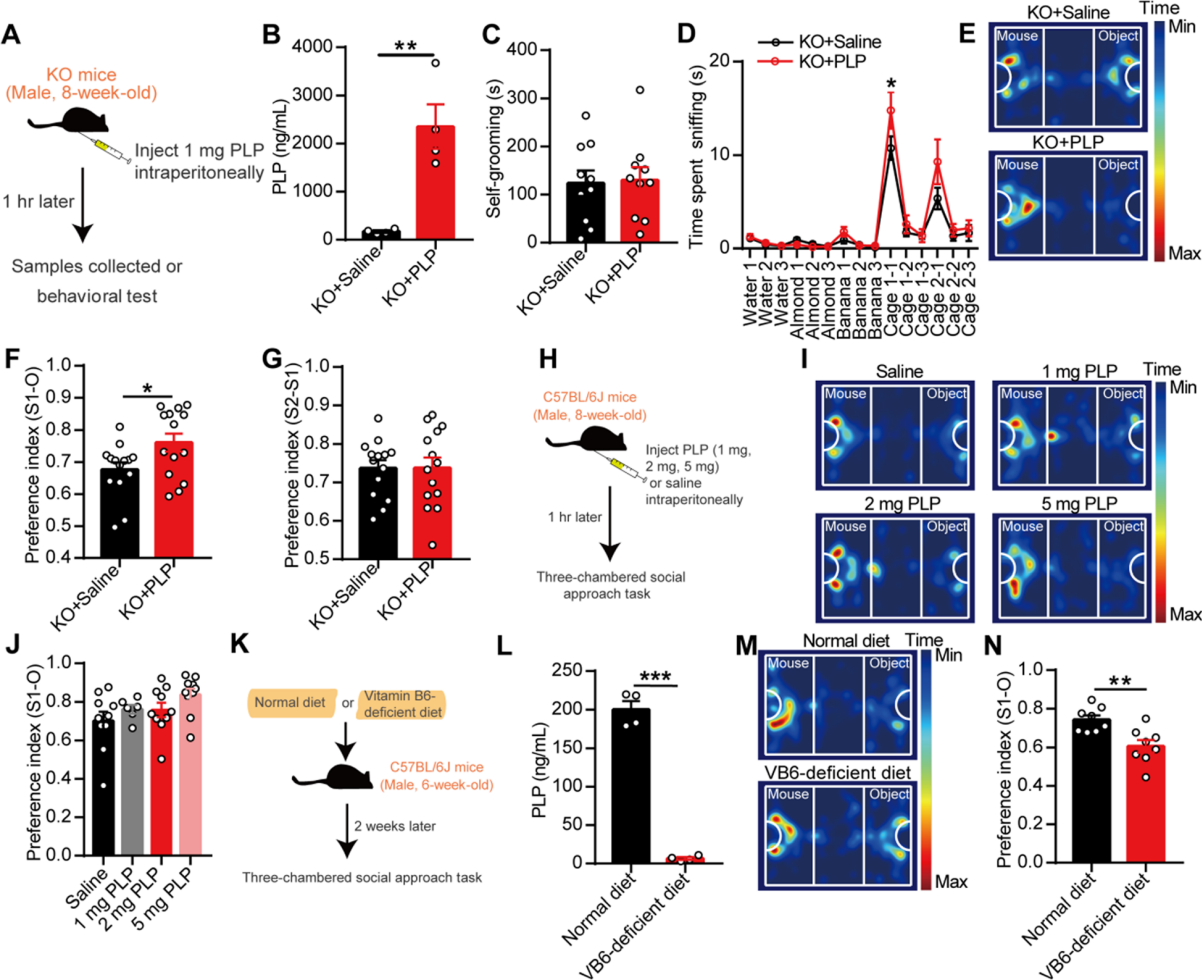
Gut microbiota-mediated vitamin B_6_ homeostasis regulated social behavior in EphB6-deficient mice. **a**–**g** Schematic of the injection of PLP (**a**). The 8-week-old male KO mice were injected with 1 mg PLP or saline intraperitoneally. After 1 h, mice were either sacrificed to detect PLP in plasma (**b**, *n* = 4 mice for each group) or subjected to self-grooming test (**c**, *n* = 10 mice for each group), olfactory habituation/dishabituation test (**d**, *n* = 8, 9 mice respectively), and three-chambered social approach task (**e**–**g**, *n* = 14 mice for each group). Different mice were used for different behavioral tests. **h**–**j** Schematic of the injection of PLP (**h**). Saline or PLP (1 mg, 2 mg, 5 mg per 0.2 mL) were injected intraperitoneally to 8-week-old male C57BL/6J mice. After 1 h, three-chambered social approach task was conducted (**i**, **j**, *n* = 10, 6, 10, 10 mice respectively). **k**–**n** Schematic of deficiency of vitamin B_6_ in 6-week-old SPF male C57BL/6J mice (**k**). Normal diet contained 12 mg vitamin B_6_ and vitamin B_6_-deficient diet were provided for 6-week-old C57BL/6J mice for 2 weeks. The level of PLP in plasma of C57BL/6J was detected (**l**, *n* = 4 mice for each group) and three-chambered social approach task (**m**, **n**, *n* = 8, 8 mice respectively) were conducted. Data shown are mean ± SEM. Two-tailed unpaired student’s *t* test (**b**, **c**, **f**, **g**, **l**, **n**), one-way ANOVA (**j**), mixed design ANOVA with genotype as independent factor and stimuli/trials as repeated-measure factor (**d**). *p < 0.05; **p < 0.01; ***p < 0.001. *WT* EphB6^+/+^ mice, *KO* EphB6^−/−^ mice, *PLP* pyridoxal 5′-phosphate, *VB6* vitamin B_6_. Statistical values are presented in Additional file 3: Table S2

We subsequently supplied vitamin B_6_ to the KO mice to clarify its effect on autism-like behavior. However, intragastric supplementation with vitamin B_6_ did not ameliorate the social deficits in the KO mice (Additional file 1: Figure S7a-c). One hour after the intraperitoneal injection of 1 mg PLP, the KO mice presented higher levels of PLP in plasma (Fig. 6b) and increased social behavior (Fig. 6d–f) compared with the control mice. No changes in self-grooming (Fig. 6c) and social novelty (Fig. 6g) were detected in the KO mice after the injection of PLP. Additionally, the intraperitoneal injection of 1 or 2 mg of PLP exerted no effect on the social behavior of C57BL/6J mice (Fig. 6h–j). Moreover, after being fed without vitamin B_6_ for 2 weeks, C57BL/6J mice presented lower plasma PLP levels and decreased social behavior (Fig. 6k–n). Conclusively, our results proved the existence of a relationship between gut microbiota-mediated defects of vitamin B_6_ and social deficits in EphB6-deficient mice.

### Gut microbiota-mediated vitamin B_6_ homeostasis regulated dopamine in the PFC of EphB6-deficient mice

**Fig. 7:**
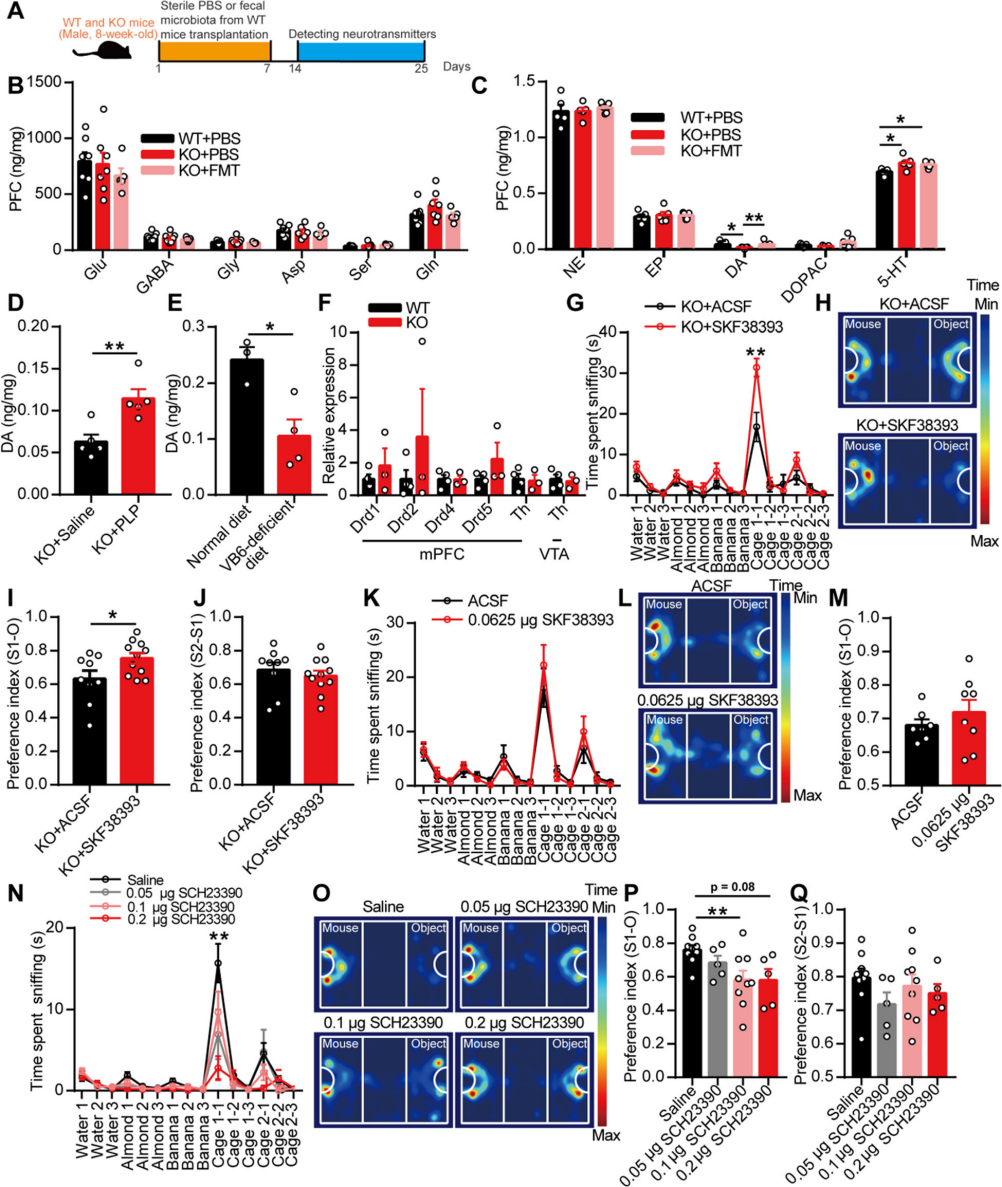
The modulated dopamine by gut microbiota-mediated vitamin B_6_ regulated social behavior of EphB6-deficient mice. **a**–**c** The 8-week-old male WT and KO mice were orally gavaged with the fecal microbiota of 8-week-old male WT mice or sterile PBS for 1 week (**a**). After 1 week, the level of amino acid neurotransmitters (**b**, *n* = 7, 7, 5 mice respectively) and monoamine neurotransmitters (**c**, *n* = 5, 5, 7 mice respectively) in PFC of mice were detected. **d** One hour after the injection of 1 mg PLP intraperitoneally, the level of DA in PFC of 8-week-old male KO mice was increased compared with that of KO mice injected with saline. *n* = 5 mice for each group. **e** The level of DA in PFC of SPF male C57BL/6J mice fed without vitamin B_6_ was decreased compared with that of C57BL/6J mice fed with normal diet. *n* = 3, 4 mice respectively. **f** The mRNA expression of dopamine receptors and Th in mPFC or VTA of 8-week-old WT and KO mice were detected by qRT-PCR. *n* = 4, 3 mice respectively. **g**–**j** Thirty minutes after the injection of D1R agonist (SKF38393, 0.0625 μg/0.3 μL) in mPFC of 8-week-old male KO mice, olfactory habituation/dishabituation test (**g**) or three-chambered social approach task (**h**–**j**) was conducted with an interval of 1 week. *n* = 9, 11 mice respectively. **k**–**m** The 8-week-old SPF male C57BL/6J mice that injected with D1R agonist (SKF38393, 0.0625 μg/0.3 μL) in mPFC were performed with olfactory habituation/dishabituation test (**k**) or three-chambered social approach task (**l**, **m**) with an interval of 1 week. *n* = 7, 8 mice respectively. **n**–**q** The 8-week-old SPF male C57BL/6J mice were injected with D1R antagonist (SCH23390) in mPFC, and olfactory habituation/dishabituation test (**n**, *n* = 8, 8, 9, 8 mice respectively) or three-chambered social approach task (**o**–**q**, *n* = 9, 5, 9, 5 mice respectively) were conducted with an interval of 1 week. Data shown are mean ± SEM. Two-tailed unpaired student’s *t* test (**d**–**f**, **i**, **j**, **m**), one-way ANOVA (**b**, **c**, **p**, **q**), mixed design ANOVA with genotype as independent factor and stimuli/trials as repeated-measure factor (**g**, **k**, **n**). *p < 0.05; **p < 0.01. *WT* EphB6^+/+^ mice, *KO* EphB6^−/−^ mice, *FMT* fecal microbiota transplantation, *PBS* phosphate-buffered saline, *PFC* prefrontal cortex, *PLP* pyridoxal 5′-phosphate, *VB6* vitamin B_6_, *Glu* glutamic acid, *GABA* gamma-aminobutyric acid, *Gly* glycine, *Asp* aspartic acid, *Ser* serine, *Gln* glutamine, *NE* norepinephrine, *EP* epinephrine, *DA* dopamine, *5*-*HT* 5-hydroxytryptamine, *DOPAC* dihydroxy-phenyl acetic acid, *mPFC* middle prefrontal cortex, *VTA* ventral tegmental area, *Th* tyrosine hydroxylase, *ACSF* artificial cerebrospinal fluid. Statistical values are presented in Additional file 3: Table S2

Vitamin B_6_, as a co-factor, has been implicated in more than 140 biochemical reactions in cells, including the biosynthesis and catabolism of amino acids and neurotransmitters [32]. As the most important active substances in the brain, we first detected neurotransmitters in the PFC of mice by high-performance liquid chromatography (HPLC) and found similar levels of glutamate, gamma-aminobutyric acid (GABA), glycine, aspartic acid, serine, and glutamine in the WT and KO mice gavaged with sterile PBS or the fecal microbiota from the WT mice (Fig. 7a, b). Interestingly, the PFC of the KO mice exhibited decreased dopamine levels and increased 5-hydroxytryptamine (5-HT) levels than that of the WT mice (Fig. 7c). Treatment with the fecal microbiota from the WT mice increased the level of dopamine but did not affect the level of 5-HT in the PFC of the KO mice compared with the levels found in the KO mice gavaged with sterile PBS. The levels of noradrenaline, epinephrine, and dihydroxy-phenyl acetic acid (DOPAC) did not differ among the three groups of mice (Fig. 7c). More excitingly, the level of dopamine in the PFC of SPF C57BL/6J mice gavaged with the fecal microbiota from the KO mice was lower than that in the PFC of C57BL/6J mice gavaged with the fecal microbiota from the WT mice (Additional file 1: Figure S8a-e). Additionally, the intraperitoneal injection of PLP increased the level of dopamine in the PFC of the KO mice (Fig. 7d), and vitamin B_6_ deficiency decreased the level of dopamine in the PFC of SPF C57BL/6J mice (Fig. 7e). Briefly, these results indicated that gut microbiota-mediated vitamin B_6_ homeostasis could affect the level of dopamine in the PFC of mice.

To determine whether the decrease in dopamine contributed to the autism-like behavior of EphB6-deficient mice and considering the fast metabolism of dopamine in the brain, we injected agonists of dopamine receptors into the mPFC of mice. The deletion of EphB6 had no effect on the mRNA expression of dopamine receptors or tyrosine hydroxylase (Th) in the mPFC or ventral tegmental area (VTA) (Fig. 7f). We then injected an agonist of dopamine D1 receptor (D1R) (SKF38393) or dopamine D2 receptor (D2R) (quinpirole) into the mPFC of mice. The results showed that the KO mice exhibited increased social behavior (Fig. 7g–j) after injection with SKF38393 compared with the KO mice injected with artificial cerebrospinal fluid (ACSF). However, no differences were found between C57BL/6J mice injected with ACSF and C57BL/6J mice injected with SKF38393 (Fig. 7k–m). In contrast, quinpirole did not increase social behavior in the KO mice (Additional file 1: Figure S8f-h), and the D1R antagonist induced decreased social behavior in C57BL/6J mice (Fig. 7n–q). In short, these results indicated that dysregulated gut microbiota and vitamin B_6_ defect led to autism-like behavior via the D1R-mediated pathway in EphB6-deficient mice.

### Gut microbiota regulated the E/I balance in the mPFC of EphB6-deficient mice

It is generally thought that D1Rs modulate GABAergic inhibition in the PFC [33]. Additionally, an E/I imbalance in synaptic transmission and neural circuits has been implicated in ASD [34–36]. Moreover, correction of the E/I imbalance can normalize key autistic phenotypes in animal models of ASD [37].

**Fig. 8:**
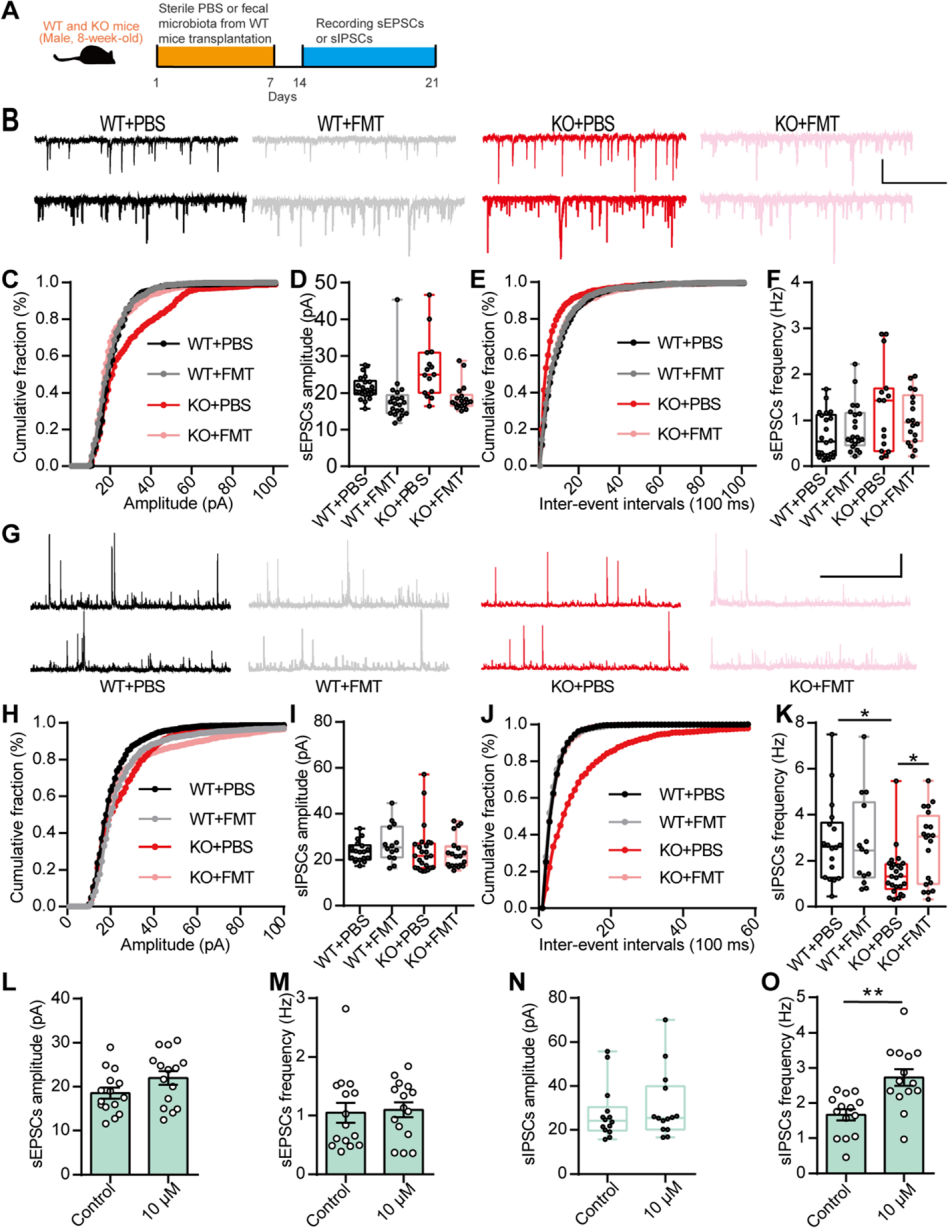
Gut microbiota and dopamine modulated E/I balance in mPFC of EphB6-deficient mice. **a** The 8-week-old male WT and KO mice were orally gavaged with the fecal microbiota from 8-week-old male WT mice or sterile PBS for 1 week. After 1 week, we recorded sEPSCs (**b**–**f**) and sIPSCs (**g**–**k**) of pyramidal neurons in mPFC of mice. **b**–**f** Representative sEPSCs traces from pyramidal neurons in mPFC of mice (**b**) were presented, scale bars: 3 s, 20 pA. Cumulative distribution of sEPSCs amplitudes (**c**), average amplitude of sEPSCs (**d**), cumulative distribution of sEPSCs frequencies (**e**), and average frequency of sEPSCs (**f**) were showed. *n* = 22, 21, 15, 18 cells from at least four mice respectively. **g**–**k** Representative sIPSCs traces from pyramidal neurons in mPFC of mice (**g**) were presented, scale bars: 3 s, 20 pA. Cumulative distribution of sIPSCs amplitudes (**h**), average amplitude of sIPSCs (**i**), cumulative distribution of sIPSCs frequencies (**j**), and average frequency of sIPSCs (**k**) were showed. *n* = 18, 14, 25, 20 cells from at least four mice respectively. **l**–**o** The mPFC slices of 8-week-old male KO mice were treated with 10 μM D1R agonist, then sEPSCs and sIPSCs of pyramidal neurons were recorded. Average amplitude (**l**) and frequency (**m**) of sEPSCs and average amplitude (**n**) and frequency (**o**) of sIPSCs were showed. *n* = 15, 15 or 14, 14 cells from at least four mice respectively. Data shown are mean ± SEM or median ± IQR. Two-tailed unpaired student’s *t* test (**l**, **m**, **o**), Mann Whitney test (**n**), Kruskal-Wallis test (**d**, **f**, **i**, **k**). *p < 0.05; **p < 0.01. *WT* EphB6^+/+^ mice, *KO* EphB6^−/−^ mice, *FMT* fecal microbiota transplantation, *PBS* phosphate-buffered saline, *mPFC* middle prefrontal cortex, *sEPSCs* spontaneous excitatory postsynaptic currents, *sIPSCs* spontaneous inhibitory postsynaptic currents. Statistical values are presented in Additional file 3: Table S2

**Fig. 9:**
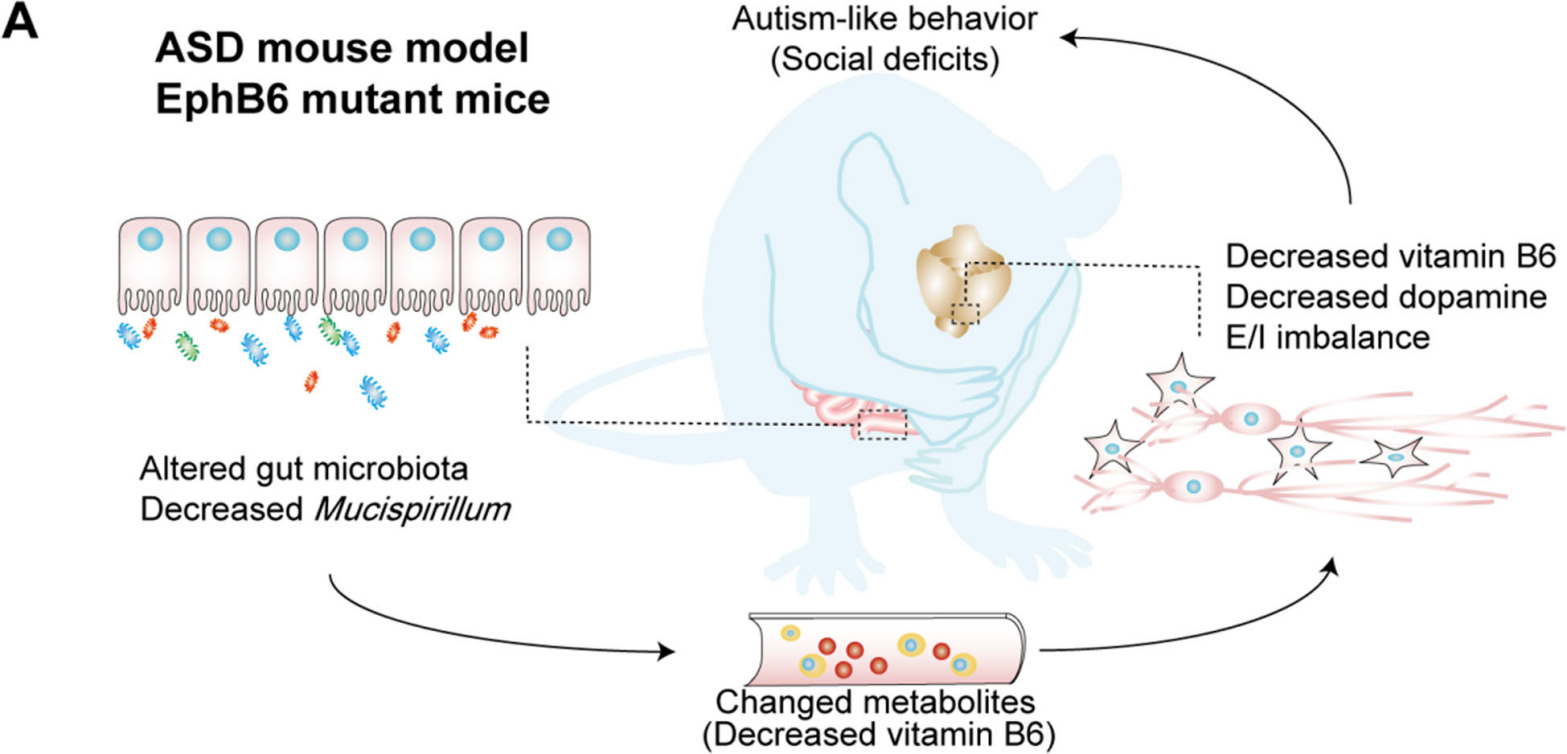
Working model of the modulated social deficits in EphB6-deficient mice by gut microbiota. **a** Deletion of EphB6 induced gut microbial dysbiosis and decreased vitamin B6 in plasma and PFC, which led to the decreased dopamine, E/I imbalance and social deficits in mice.

To further investigate the cellular mechanism underlying the gut microbiota-mediated autism-like behavior in EphB6-deficient mice, we recorded spontaneous excitatory postsynaptic currents (sEPSCs) and spontaneous inhibitory postsynaptic currents (sIPSCs) of mPFC pyramidal neurons in WT and KO mice treated with sterile PBS or the fecal microbiota from the WT mice (Fig. 8a). The amplitude and frequency of the sEPSCs did not differ among the groups (Fig. 8b–f). The amplitude of sIPSCs also showed similarities among the groups, whereas the frequency of sIPSCs was decreased in the KO mice and was rescued by transplantation of the fecal microbiota from the WT mice (Fig. 8g–k). Additionally, we found a decreased frequency of sIPSCs in pyramidal neurons of the mPFC of C57BL/6J mice gavaged with the fecal microbiota of the KO mice (Additional file 1: Figure S9a-j). The D1R agonist, at a concentration of 10 μM, increased the frequency of sIPSCs in pyramidal neurons of the mPFC of KO mice (Fig. 8l–o), whereas the same concentration of the D1R agonist had no effect on sEPSCs or sIPSCs recorded in pyramidal neurons of the mPFC of the WT mice (Additional file 1: Figure S9k-n). Collectively, these results indicated that the gut microbiota modulated the E/I balance, which was likely regulated by dopamine, in pyramidal neurons of the mPFC of EphB6-deficient mice (Fig. 9).

## Discussion

Increasing evidence, particularly from clinical studies of ASD patients, suggests a functional link between the gut microbiota and the development of ASD. In addition, genomic and transcriptome studies of ASD patients have revealed many candidate genes for ASD. However, the functions of ASD-associated genes and the mechanisms linking the gut microbiota and brain dysfunctions (gut-brain axis) in ASD have not been fully elucidated.

First, our study revealed that EphB6 is functionally an ASD-associated gene. *EPHB6* has been considered a candidate gene for ASD for a long time [15–17] and is downregulated in ASD patients [19, 20]. Here, using our transgenic mouse models, we found that the deletion of EphB6 in mice induced autism-like behavior that mimicked the core symptoms of ASD patients fairly well. Through whole-genome sequencing and transcriptome analysis, researchers have identified more than 1000 genes that are associated with ASD, including *EPHB6* [15–18], *EPHA1* [15], and *EPHB2* [38]. Our study uncovers the functional role of EphB6 in ASD and suggests that EphB6-deficient mice can be used as a new mouse model of ASD.

Second, we found that gut microbial dysbiosis is required for autism-like behavior in EphB6-deficient mice. Partial behavioral phenotype could also be transferred to abx-treated C57BL/6J mice by transplantation of fecal microbiota from KO mice. Data on abx-treated C57BL/6J mice further confirm the important role of gut microbiota on autism behavior, although antibiotic treatment causes multiple effects and brain injury. Most ASD patients exhibit serious GI problems [5, 6, 26] and a changed gut microbiota composition [39, 40]. Moreover, microbiota transfer therapy can improve GI and autistic symptoms in ASD children [11, 12]. Our study also suggests the probable role of the gut microbiota in treating the core symptoms of ASD in adult patients. Eph families play an important role in regulating epithelial homeostasis by interacting with epithelial cell adhesion and junction proteins [22]. Cldn4 can interact with EphA2 and ephrin-B1 to affect tight junction integration [41, 42]. AF-6 can be recruited to cell–cell contacts in MDCK and 293T cells by interacting with Eph receptors, including EphB6 [43, 44]. The ablation of EphB6 might induce the dysregulated interaction between Eph families and junction proteins that leads to increased intestinal mucosal permeability and gut microbial dysbiosis in mice.

Third, we found that defect of vitamin B_6_ is crucial for the gut microbiota-mediated autism-like behavior in EphB6-deficient mice. In addition, the decreased level of vitamin B_6_ in the plasma and PFC of EphB6-deficient mice was rescued by transplantation of the fecal microbiota from the WT mice. Moreover, the intraperitoneal injection of vitamin B_6_ rescued the social deficits of EphB6-deficient mice. More interestingly, PLP has been detected at unbelievably lower levels in ASD children than in control children [45]. Many clinical studies from the 1960s treated ASD children with vitamin B_6_, and most of these studies reported improvements in their autistic symptoms [46–48]. Previous studies have also reported that vitamin B_6_ does not have an effect on ASD patients [49]. Considering the complicated causes of ASD, we hypothesize that vitamin B_6_ is effective for some ASD patients, such as ASD patients with downregulated *EPHB6* expression. Vitamin B_6_ cannot be synthesized by the body itself, and vitamin B_6_ in the body is mainly obtained from diet and bacterial synthesis via intestinal absorption. Therefore, normal intestinal functions are important for the homeostasis of vitamin B_6_ in the body. The intestinal absorption of vitamin B_6_ is dependent on the pH, and a higher uptake is observed with acidic compared with alkaline pH values [50]. The more alkaline environment in the gut of EphB6-deficient mice (Additional file 1: Figure S1i) might cause decreased absorption of vitamin B_6_, but how the changed bacterial composition affects the gut pH and vitamin B_6_ levels in feces and blood requires further exploration. Overall, our study found a new role for the gut microbiota on the modulation of vitamin B_6_ and confirmed that gut microbiota-mediated vitamin B_6_ ameliorated the social deficits of EphB6-deficient mice.

Fourth, we functionally established the mechanisms linking the gut microbiota and brain dysfunctions (gut-brain axis) in EphB6-deficient mice. The gut-brain axis is generally considered to be involved in psychiatric diseases. However, few studies have investigated how the brain is specifically regulated by the gut microbiota. In our study, we found that the dopamine level in the PFC of EphB6-deficient mice was regulated by gut microbiota-mediated vitamin B_6_. ASD patients exhibit lower medial prefrontal dopaminergic activity [51]. After the administration of vitamin B_6_, autistic children exhibit reduced levels of urinary homovanillic acid, which suggests an improved dopamine metabolism [52]. These studies indicate that vitamin B_6_ regulates dopaminergic metabolites in ASD patients. Sgritta previously reported that VTA plasticity is modulated by *Lactobacillus reuteri* in ASD mouse models [10]. Here, our study showed a new regulatory role for the gut microbiota on dopamine in the PFC by modulating vitamin B_6_ in EphB6-deficient mice. Moreover, we found that D1R agonists ameliorates the social deficits of EphB6-deficient mice and the gut microbiota-modulated E/I imbalance in the mPFC of these mice. The activation of D1Rs in the PFC can increase the frequency of sIPSCs in pyramidal neurons, whereas D2R agonists do not exert the same effect [33]. The modulation of social behavior by D1Rs was likely due to its modulation of GABAergic inhibition in EphB6-deficient mice. Collectively, the results from our study indicated that decreases in the level of dopamine were induced by dysregulated gut microbiota-mediated defects in vitamin B_6_ and then contributed to E/I imbalance and social deficits in EphB6-deficient mice.

## Conclusions

In summary, our study uncovers a key role for the gut microbiota in the autism-like behavior of EphB6-deficient mice. Mechanistically, gut microbiota-mediated defects in vitamin B_6_ regulate autism-like social behavior by decreasing the dopamine levels and inducing E/I imbalance in the mPFC of EphB6-deficient mice. Our study suggests a new ASD mouse model, proves the important role of the gut microbiota in genetic factor-induced autism, and provides new insight into the gut-brain-microbiota axis.

## Methods

### Mice

All the mice used in our experiments were male. SPF C57BL/6J mice (3–8 weeks old) were obtained from Animal Experiment Center of Southern Medical University in Guangzhou of China. EphB6-deficient mice were generated using the embryonic stem (ES) cells with the insertion of EphB6^tm1e(KOMP)Wtsi^ vector (IKMC project number: 49365). The injection of ES cells and obtaining of chimeric mice were operated by subsidiary of Cyagen Biosciences Inc. in Guangzhou of China. The chimeric mice were crossed with SPF C57BL/6J mice and its offspring (EphB6^+/−^ mice) were kept being crossed with SPF C57BL/6J mice until the fifth generation was born. Then EphB6^+/−^ mice were crossed with each other to obtain EphB6^+/+^ mice and EphB6^−/−^ mice. All the mice were raised at a controlled appropriate SPF condition with the temperature at 24 ± 1 °C and humidity at 50% to 70% and with 12-h light-dark cycles by turning lights on from 8:00 a.m. to 8:00 p.m.. The animals were housed in groups of 4–5 in plastic cages (Exhaust Ventilated Closed-System Cage Rack). Standard sterile diet and drinking water for raising mice were used to feed the mice. The EphB6 ablation mice were genotyped at 2-week-old using the primers 5′-CTCTGCCAAGGTGAGACACTTTTCC-3′ and 5′-AGCCAGTCTCTACCTCCTGTTTTGG-3′ for the wild-type band, 5′-CTCTGCCAAGGTGAGACACTTTTCC-3′ and 5′-CGTGGTATCGTTATGCGCCT-3′ for the mutant band and weaned at 3-week-old. All procedures treated with mice were in compliance with the Regulations for the Administration of Affairs Concerning Experimental Animals in China.

### Fecal microbiota transplantation

Fresh feces of healthy male EphB6^+/+^ and EphB6^−/−^ mice (eight mice for each group from at least three cages) were collected from the disinfected anus and then put into new sterile tubes every day before the experiment, which would maintain microbial vitality [4]. Then the fresh feces were weighted, mixed with sterile PBS at a dilution ratio of 1 mg/10 μL or 1 mg/20 μL, and centrifuged at 900×*g* for 3 min. The supernatant was collected and gavaged orally to each mouse (10 mL/kg) for five or seven consecutive days. All the mice were handled aseptically. Not specifically mentioned, in most of our experiments, fecal microbiota transplantation (FMT) experiments were done according to this protocol.

The dilution ratio of 1 mg/20 μL was used to treat 3-week-old SPF C57BL/6J mice and the dilution ratio of 1 mg/10 μL was used to treat 6-week-old SPF C57BL/6J mice and 8-week-old EphB6^+/+^ and EphB6^−/−^ mice.

For fecal metabolites or fecal microbiota excluding metabolites transplantation, after the fresh feces were weighted, mixed with sterile PBS at a dilution ratio of 1 mg/10 μL and centrifuged at 4000×*g* for 10 min, the supernatant and precipitate were both collected. After being filtered by the filter with a pore size of 0.22 μm (Cat# SLGP033RS, Millipore, Darmstadt, Germany), the supernatant was orally gavaged to 6-week-old SPF C57BL/6J mouse (10 mL/kg) for seven consecutive days (Fig. 3j–m). And the precipitate, which contained microbiota, was re-suspended in sterile PBS and centrifuged at 900×*g* for 3 min. After that, the supernatant was collected, washed by sterile PBS twice to exclude the metabolites, and orally gavaged to 6-week-old SPF C57BL/6J mouse (10 mL/kg) for seven consecutive days (Additional file 1: Figure S5j-m).

### Antibiotic treatment

Vancomycin (50 mg/kg, CAS: 123409-00-7, MP bio, California, USA), neomycin (100 mg/kg, CAS: 1405-10-3, MP bio), and metronidazole (100 mg/kg, CAS: 443-48-1, MCE, New Jersey, USA) were mixed using sterile drinking water [4]. Then the mixture was orally gavaged to 3-week-old or 6-week-old SPF C57BL/6J mice twice a day for five or seven consecutive days and the amount of infusion was based on the weight of mice. The mixture was prepared every day and used freshly. During the treatment, ampicillin (1 mg/mL, CAS: 69-52-3, MP bio) was added into the drinking water of mice and changed with fresh solution every 3 days. For 3-week-old SPF C57BL/6J mice, the antibiotic treatment lasted for 5 days [53]. All the mice were handled aseptically.

### Western blot analysis

After abdominally anesthetized with phenobarbital sodium (60 mg/kg), the brain of mouse was quickly removed, put into an ice-cold mouse brain mold (Cat# 68713, RWD, Shenzhen, China), and sliced. Then posterior mPFC, hippocampus, and cerebellum of mice were collected. The total proteins of tissues were extracted using the lysis buffer (Cat# P0013B, Beyotime, Shanghai, China) and boiled in protein loading buffer. Equal amounts of the denatured protein samples were electrophoresed in 6–10% polyacrylamide gel containing 0.1% SDS and transferred to polyvinylidene fluoride (PVDF) membranes with a pore size of 0.45 μm (Cat# IPVH00010, Millipore). When transferring the protein in the polyacrylamide gel to the PVDF membrane, polyacrylamide gel, PVDF membrane, and fliter paper were stacked together and put into the transfer clip. Then the clip was put into transfer tank and the protein in polyacrylamide gel was transferred to the membrane at 250 mA for 2 h. The PVDF membranes were incubated with primary antibodies at 4 °C for at least 12 h, and then were incubated with secondary antibodies for about 2 h at room temperature (Cat# BA1050 and Cat# BA1054, Boster, California, USA). Equal volume of Clarity Western Peroxide Reagent and Clarity Western Luminol/Enhancer Reagent (Cat# 1705060, Biorad, California, USA) were mixed together. The mixed reagent was added onto the membrane and the desired signals were visualized by Quantitative FluorChem SP Imaging System (Alpha Innotech, California, USA). Integrated density of each band was measured by ImageJ software and values of the corresponding band of Gapdh were considered as internal controls. Then the integrated density of Gapdh of control mice was normalized to 1 and the integrated density of other band was compared with that of Gapdh. The following primary antibodies were used: EphB6 (1:500, Cat# ab54656, Abcam, Cambridge, UK), c-Fos (1:500, Cat# MABE329, Millipore), and Gapdh (1:5000, Cat# 60004-1-ig, Proteintech, Chicago, USA).

### Quantitative reverse transcription PCR

After anesthetized with phenobarbital sodium (60 mg/kg), different tissues of mice were quickly removed and put into liquid nitrogen, including colon, colonic epithelium, spleen, and lung. Then posterior mPFC and VTA were sectioned out using ice-cold mouse brain mold (Cat# 68713, RWD). Quantitative reverse transcription PCR (qRT-PCR) was performed accordingly [54] by using a 7500 real-time PCR system (ABI, California, USA) and SYBR Premix Ex Taq (Cat# RR420A, Takara, Osaka, Japan). Normalized to the mRNA expression level of Gapdh or Actb, the mRNA expression of other genes was evaluated using the method of ΔΔCt. All primers used in qRT-PCR were listed in Additional file 2: Table S1.

### Hematoxylin-eosin staining

Hematoxylin and eosin staining of different tissues of mice was performed accordingly [54]. Briefly, the fresh tissues were immersed into 4% formaldehyde immediately for 24 h. Then tissues were embedded in paraffin, sectioned, and stained with hematoxylin and eosin. The stained sections were observed using an optical microscope (Olympus, Tokyo, Japan). Morphological characteristics were analyzed in a blinded manner by a specialized pathologist. To evaluate histological changes of intestines [55], inflammatory infiltrate and epithelial injury were both scored. Inflammatory infiltrate was scored according to severity of infiltrate and area involved lymphocytes, neutrophils, plasma cells, or eosinophils. Epithelial injury was scored according to area of section, mucodepletion of glands, intraepithelial lymphocytes, and ulcer/erosion. Histopathological changes were scored on a scale of 0–3 (where 0 = none; 1 = mild; 2 = moderate; 3 = severe) for each parameter. Area involved was scored as follows: 0 = no involvement; 1 = < 25% of section; 2 = < 50%; 3 = < 75%; 4 = < 100%. The maximum of inflammatory infiltrate score was 7. The maximum of epithelial injury score was 13. To evaluate histological changes of lung [56], the parameters for bronchitis, edema, interstitial inflammation, and intraalveolar inflammation were also graded on a scale of 0 to 3. The maximum of lung inflammation score was 12. To evaluate histological changes of liver [56], inflammation, necrosis/abscess formation, and thrombus formation were also graded on a scale of 0 to 3. The maximum of liver inflammation score was 9.

### Intestinal permeability assay

Mice were fasted for 4 h before the experiment, then FITC-dextran (50 mg/mL, Cat# 46944, Sigma Aldrich, Missouri, USA) was gavaged to mice (600 mg/kg) [8]. After 4 h, the blood of the mouse was collected by cardiac puncture and was placed at room temperature for 1 h before being centrifuged at a speed of 3000 rpm for 10 min. Then the supernatant was transferred to a new tube and centrifuged at a speed of 12,000 rpm for 10 min at 4 °C. The supernatant, which was the serum, was diluted with equal volume of PBS and 100 μL diluted serum was added to a 96-cell microplate. The concentration of FITC in serum was determined by Varioskan LUX microplate reader (Thermo Fisher Scientific, Massachusetts, USA) with an excitation of 485 nm and an emission wavelength of 528 nm. The serial diluted FITC-dextran (0, 0.5, 1, 2, 4, 6, 8, 10 μg/μL) was used as standards. Serum of mice administered with PBS was used as negative controls.

### Vitamin B_6_-deficient mouse model

The formula of diet with normal vitamin B_6_ or without vitamin B_6_ was based on previous study [57]. Then 6-week-old SPF male C57BL/6J mice were fed with the diet with or without vitamin B_6_ for 2 weeks.

### Behavioral studies

Mice used in behavioral experiments were male and naive. Mice were handled everyday for 3 days before the experiments and habituated in the experiment room for at least 30 min before each test [58]. Mice were performed with different behavioral tests with a sequence or different mice were used for different behavioral tests which were mentioned in the figure legends. The sequence of different behavioral tests was self-grooming test, olfactory habituation/dishabituation test, three-chambered social approach task, marble burying test, open field test, social partition test, elevated plus maze, and Morris water maze. Different behavioral tests were done with an interval of at least 2 days.

Self-grooming test was performed as previously described [59]. Generally, mouse was placed in an empty crystal cage to habituate the cage for 10 min and then the time each mouse spent self-grooming was recorded during the next 10 min by a double-blind experienced experimenter. Self-grooming included face-wiping, scratching/rubbing of head and ears, and full-body grooming. Between each trial, the apparatus was cleaned by 30% ethyl alcohol in water.

Marble burying test was performed as previously described [60]. Mouse was placed into an animal cage filled with fresh wood chip bedding with the depth of 5 cm. Regular pattern of glass marbles (five rows of four marbles), which were placed 4 cm apart from each other, were regularly placed under the bedding. Mouse was allowed to explore the cage for 30 min. The number of buried (more than 50% of their depth in bedding) marbles was counted.

Social partition test was performed as previously described [61]. The experimental mice were housed individually for 4 days before the partition test. On the day before the test, the sex- and age-matched C57BL/6J partner mice were placed on the opposite side of the transparent partition with 0.6-cm-diameter holes. This C57BL/6J mouse was considered as the familiar mouse. At the first trial, the total time spent at partition for the experimental mouse during 5 min was recorded. Then the familiar mouse was replaced with another sex- and age-matched unfamiliar C57BL/6J mouse, the total time spent at partition during 5 min was recorded. In the last trial, the unfamiliar mouse was instead replaced by the familiar mouse and the time spent at partition during 5 min was recorded. The time spent at partition in the three trials was recorded by a double-blind experienced experimenter.

Olfactory habituation/dishabituation test was performed as previously described [62]. Mouse was placed into a clean usual animal cage with thin bedding and habituated to the fresh room for 30 min before test. One swab saturated with water was given to mouse for 2 min, then quickly replaced by another swab saturated with water for the following 2 min, and then third swab saturated with water was given to mice for another 2 min quickly. The other odors were presented to mouse similarly. The sequence of presented odors was water, almond extract, imitation banana flavor, odor of soiled bedding from one cage of C57BL/6J mice, and odor of soiled bedding from another different cage of C57BL/6J mice. Water, almond extract (dilution of 1:100), and imitation banana flavor (dilution of 1:100) were regarded as unsocial odors, while soiled bedding with the excrement of sex- and age-matched unfamiliar C57BL/6J mice were regarded as social odors. To prepare social odors, C57BL/6J mice would live in the cage for at least 3 days. Then cotton swabs were used to wipe in a zig-zag pattern across the bottom surface of the cage and would be stained with the soiled bedding. The time each mouse spent sniffing the odorant swabs in every 2-min trial was recorded by a double-blind experienced experimenter.

Three-chambered social approach task was performed as previously described [63]. The apparatus was divided into three rectangular clear chambers (60 cm × 40 cm × 22 cm) by two walls on which two removable doorways (8 cm × 5 cm) were installed to allow mouse to access each chamber freely. After a habituation to the middle chamber for 5 min, the mice were allowed to explore the three chambers freely for 10 min. For the sociability test, an age- and sex-matched C57BL/6J mouse was placed in the wire cage in one chamber while the wire cage in the other chamber was empty. Then the dividers were raised and the experimental mouse was allowed to freely explore all three chambers for 10 min. For the social novelty test, another age- and sex-matched C57BL/6J mouse was placed in the empty wire cage described above. And the experimental mouse was originally placed in the center of the chamber and allowed to freely explore the chambers for 10 min after doorways were removed. Between each trial, the apparatus was cleaned by 70% ethyl alcohol in water. Time spent in each chamber and mouse trajectory were calculated using EthoVison XT software (Noldus, Wageningen, Netherlands). And the time spent sniffing the wire cages which represented the social approach behavior of mice was calculated by a double-blind experienced experimenter.

In open field test, the mouse was placed in the center of an open field chamber (40 cm × 40 cm × 30 cm) [64]. Exploratory behavior of mice was assessed by a session of 30 min and total distance was automatically recorded and analyzed by a VersaMax animal behavioral monitor system (Omnitech Electronics, Nova Scotia, Canada).

Elevated plus maze test was performed accordingly [64]. The elevated plus maze was consisted of two opposing open arms (30 cm × 5 cm × 0.5 cm), two opposing enclosed arms (30 cm × 5 cm × 15 cm), and a central platform (5 cm × 5 cm). The mouse was put into the center of the maze for 5 min and the time spent in different arms and entries into different arms were recorded by EthoVison XT software (Noldus).

Morris water maze test was performed as before [65]. Generally, four trials were given to each mouse every day for 5 days. In each trial, the searching time for the mouse was no more than 1 min. A stay on the platform was 15 s. Intervals between each trial were no less than 1 min. On the sixth day, the probe test was performed by removing the platform and the swimming paths of mice in 1 min were recorded. The swimming paths of mice during the learning and test period were analyzed by EthoVison XT software (Noldus).

### 16S rRNA gene sequencing

Fecal samples of the experimental mice were collected and stored at − 80 °C before being performed. Using QIAamp Fast DNA Stool Mini kit (Cat# 51604, QIAGEN, Venlo, Netherlands), genomic DNA of samples were extracted. The purity and concentration of the extracted DNA were detected using agarose gel electrophoresis. Bacterial DNA was amplified with the primers targeting V3–V4 regions (5′-TACGGRAGGCAGCAG-3′, 5′-GGGTATCTAATCCT-3′). Then DNA was sequenced using MiSeq PE300 platform (Illumina, California, USA) by OE Biotechnology company in Shanghai. The raw data were treated and processed using QIIME software package (version 1.8.0). Then represent sequences of OTU were blasted in Silva database (version 123). The alpha diversity and beta diversity were analyzed using QIIME software package (version 1.8.0).

### Metabolomic analysis

For non-targeted metabolite analysis, PFC of mice were prepared and deproteinized with methanol. Then the samples were analyzed using liquid chromatography-mass spectrometry by oe biotechnology company in shanghai. UPLC-Q-TOF/MS (ACQUITY UPLC I-Class, Waters, Massachusetts, USA) and ESI-QTOF/MS (Xevo G2-S Q-TOF, Waters) were used. The chromatographic column was the ACQUITY UPLC BEH C18 Column (1.7 μm, 2.1 mm × 100 mm, Waters). Mobile phase A was water contained with 0.1% formic acid and mobile phase B was acetonitrile contained with 0.1% formic acid. The gradient elution was 1–5% mobile phase B in 0–1 min, 5–30% mobile phase B in 1–2 min, 30–60% mobile phase B in 2–3.5 min, 60–90% mobile phase B in 3.5–7.5 min, 90–100% mobile phase B in 7.5–9.5 min, 100% mobile phase B in 9.5–12.5 min, 100–1% mobile phase B in 12.5–12.7 min, and 1% mobile phase B in 12.7–16 min. The spectrum signal of samples was acquired by electrospray ionization using positive and negative ionization modes. The data were pretreated using progenesis QI (Waters) and then multivariate statistical analysis was performed using SIMCA software (version 14.0, Umetrics, Umeå, Sweden). The enriched pathway analysis of changed metabolites was performed using KEGG database (http://www.genome.jp/KEGG/pathway.html) and R (version 3.4.1).

For targeted metabolic analysis, PFC was pretreated with 0.4 M perchloric acid which contained 0.04% EDTA and 100 μL plasma was pretreated with 50 μL 5% trichloroacetic acid. 1 M NaOH was added to samples to quench acid.

For the analysis of amino acid neurotransmitters, high-performance liquid chromatography (HPLC, Shimadzu, Kyoto, Japan) contained with the fluorescence detection system (Prominence RF-20A/20Axs, Shimadzu) and the C18 chromatographic column (Eclipse AAA, 4.6 × 150 mm, 5 μm, Agilent, California, USA) was used. All the used reagents and liquid were chromatographically pure. Mobile phase A contained 20 mM sodium acetate solution (pH 7.2), methyl alcohol, and tetrahydrofuran which were at a volume ratio of 400:95:5. Mobile phase B contained 20 mM sodium acetate solution (pH 7.2) and methyl alcohol which were at a volume ratio of 120:380. The gradient elution was 0–63% mobile phase B in 0–10 min, 63% mobile phase B in 10–12 min, 63–100% mobile phase B in 12–12.01 min, 100% mobile phase B in 12.01–17 min, 100–0% mobile phase B in 17–18 min, and 0% mobile phase B in 18–21 min. The temperature of column was set as 35 °C. The flowing rate of mobile phase was 0.8 mL/min. The excitation wavelength was set as 340 nm, and the emission wavelength was set as 455 nm. The derivatization reagent contained o-phthalaldehyde (OPA, 5 mg, CAS: 643-79-8, Sigma-Aldrich), methyl alcohol (120 μL), β-mercaptoethanol (10 μL), and borate buffer (0.2 M, pH 9.2, 1 mL) and was kept out of light. Data were recorded by INT7. The standards, including glutamic acid (CAS: 56-86-0, Sigma-Aldrich), gamma-aminobutyric acid (CAS: 56-12-2, Sigma-Aldrich), glycine (CAS: 56-40-6, Sigma-Aldrich), aspartic acid (CAS: 56-84-8, Sigma-Aldrich), serine (CAS: 56-45-1, Sigma-Aldrich), taurine (CAS: 107-35-7, Sigma-Aldrich), and glutamine (CAS: 56-85-9, Sigma-Aldrich), were prepared at the concentrations of 7.8125, 15.625, 31.25, 62.5, 125, and 250 ng/mL. The concentrations of amino acid neurotransmitters in different samples were acquired according to that of standards.

For the analysis of monoamine neurotransmitters, HPLC contained with the electrochemical detection system (DECADE life, Antec Scientific, Zoeterwoude, Netherlands) and the chromatographic column (Accucore C18, 150 × 2.1 mm, 2.6 μm, Thermo Scientific) was used. All the used reagents and liquid were chromatographically pure. Mobile phase was prepared with deionized water and MeOH with a volume ratio of 9:1, in which NaH_2_PO_4_ (100 mM), sodium octane sulfonate (0.74 mM), Na_2_EDTA (0.027 mM), and KCl (2 mM) were contained. The temperature of column was set at 35 °C. And the flowing rate of mobile phase was set at 0.2 mL/min. The standards, including epinephrine (CAS: 51-43-4, Sigma-Aldrich), noradrenaline (CAS: 108341-18-0, Sigma-Aldrich), dopamine (CAS: 62-31-7, Sigma-Aldrich), 3,4-dihydroxyphenylacetic acid (CAS: 102-32-9, Sigma-Aldrich), homovanillic acid (CAS: 306-08-1, Sigma-Aldrich), 5-Hydroxyindole-3-acetic acid (CAS: 54-16-0, Sigma-Aldrich), and 5-hydroxytryptamine (CAS: 153-98-0, Sigma-Aldrich), were prepared at the concentrations of 0.5, 1, 25, 125, and 250 ng/mL. The concentrations of monoamine neurotransmitters in different samples were acquired according to that of standards.

For the analysis of pyridoxal 5′-phosphate, pyridoxamine, and pyridoxine, TSQ Quantiva combined with the Prelude SPLC System (Thermo Fisher Scientific) was used. All the used reagents and liquid were chromatographically pure. First, the separation of substances was performed using Prelude SPLC System with the C18 chromatographic column (Water Acquity UPLC HSS T3, 2.1 × 100 mm, 1.7 μm). Mobile phase A contained 0.2% formic acid. Mobile phase B was methyl alcohol. The gradient elution was 0–50% mobile phase B in 0–2 min, 50–95% mobile phase B in 2–3.5 min, 95% mobile phase B in 3.5–5.5 min, and 95–0% mobile phase B in 5.5–6.5 min. The temperature of column was set as 40 °C. The flowing rate of mobile phase was 0.25 mL/min. Data were recorded using positive-ion electrospray ionization and the selected reaction monitoring mode. For pyridoxal 5′-phosphate, precursor ion was *m/z* 248.03, product ion was *m/z* 150.071, and collision energy was 16.067 V. For pyridoxamine, precursor ion was *m/z* 169.152, product ion was *m/z* 152.111, and collision energy was 12.124 V. For pyridoxine, precursor ion was *m/z* 170.152, product ion was *m/z* 134.111, and collision energy was 21.073 V. Data were acquired and processed with TraceFinder software (version 3.3 sp1, Thermo Fisher Scientific). The standards, including pyridoxal 5′-phosphate (CAS: 41468-25-1, Sigma-Aldrich), pyridoxamine (CAS: 524-36-7, Sigma-Aldrich), and pyridoxine (CAS: 58-56-0, Sigma-Aldrich), were prepared at the concentrations of 0.1, 0.5, 1, 6.25, 12.5, 25, 50, and 100 ng/mL. The concentrations of pyridoxal 5′-phosphate, pyridoxamine, and pyridoxine in different samples were acquired according to that of standards.

### Bacterial culturing

PFC of mice were brought out aseptically and homogenized in PBS using sterile magnetic beads. Then the homogenate was painted on the Luria-Bertani solid medium and cultured for 24 h at 37 °C.

### DNA extraction of bacteria

The PFC of mice were cut off aseptically and the genomic DNA of the tissue was extracted using PureLink genomic DNA kit (Cat# K1820-01, Invitrogen, California, USA). Then the DNA was amplified using bacterial universal primers (5′-AGAGTTTGATCATGGCTCAG-3′, 5′-CCGGGAACGTATTCACC-3′) [66]. The DNA of *Escherichia coli* was used as positive control.

### Stereotaxic surgery and drug microinjection

The stereotaxic surgery was performed to implant brain infusion cannula into mPFC of adult male mice based on the published protocols [64]. After being anesthetized by phenobarbital sodium (60 mg/kg), the mouse was placed in a stereotaxic frame (RWD) and a hole with the diameter of 1 mm was drilled with a dental drill on the skull of mouse according to the adjusted coordinates of bilateral mPFC (AP: + 1.84 mm, ML: ± 0.4 mm, DV: − 2.2 mm). Then the brain infusion cannula (Cat# 62004, RWD) was carefully put into the drilled hole and fixed by glass ionomer cement. After the operation, the mice were resuscitated on an electric blanket and then put back into the original cage. After a recovery of 7 days, the catheter cap (Cat# 62104, RWD) was removed and the injection needle (Cat# 6220, RWD4) was inserted into the catheter after a disinfection with 75% alcohol. The injection needle was connected with a microsyringe through a polyethylene tube (Cat# 62320, RWD), and the drug was injected into the mPFC at a speed of 0.1 μL/min controlled by a microsyringe pump (Cat# R404, RWD). The total volume of the injected drug was 0.3 μL. After the injection of drug, the injection needle was kept being inserted into the catheter for 5 min to facilitate the complete diffusion and absorption of the drug. Behavioral test was performed 30 min after administration. The drugs used in this experiment were SKF38393 (CAS: 62717-42-4, MCE), quinpirole (CAS: 524-36-7, Sigma-Aldrich), and SCH23390 (CAS: 125941-87-9, MCE).

### Slice preparation

Male mice were decapitated after anesthetized by phenobarbital sodium (60 mg/kg). Brains were removed quickly and then placed into the ice-cold modified ACSF containing (in mM) 26 NaHCO_3_, 10 glucose, 10 MgSO_4_, 2 KCl, 1.3 NaH_2_PO_4_, 0.2 CaCl_2_, and 250 sucrose. Slices containing mPFC (300 μm) were prepared using a VT-1200S vibratome (Leica, Wetzlar, Germany) in ice-cold modified ACSF. Then slices were transferred into the storage chamber containing the regular ACSF (in mM) (126 NaCl, 26 NaHCO_3_, 10 glucose, 2 CaCl_2_, 3 M KCl, 1 MgSO_4_, and 1.25 NaH_2_PO_4_) at 31 °C for 1 h and then were removed to room temperature (25 ± 1 °C) for 1 h before being recorded. All solutions were saturated with 95% O_2_/5% CO_2_ (vol/vol) during the slice preparation [67].

### Electrophysiology

The neurons in mPFC were obtained using an infrared (IR)-differential interference contrast (DIC) microscope (ECLIPSE FN1, Nikon, Tokyo, Japan). To record sEPSCs, pipettes (input resistance: 3–7 MΩ) were filled with an intracellular solution containing (in mM) 105 K-gluconate, 30 KCl, 10 phosphocreatine, 10 HEPES, 4 ATP-Mg, 0.3 EGTA, and 0.3 GTP-Na (pH 7.3, 285 mOsm). When recording sEPSCs, the GABA_A_ receptors were blocked with 20 μM bicuculline methiodide (CAS: 40709-69-1, TOCRIS, Minneapolis, USA). When recording sIPSCs, the holding potentials were 0 mV, pipettes (input resistance: 3–7 MΩ) were filled with an intracellular solution containing (in mM) 110 Cs_2_SO_4_, 0.5 CaCl_2_, 2 MgCl_2_, 5 EGTA, 5 HEPES, 5 TEA, 5 ATP-Mg (pH 7.35, 285 mOsm). Data were recorded by a multiClamp 700B (Molecular Devices, San Jose, USA), digitized at 10 kHz, and filtered at 3 kHz. Data were collected when the series resistance fluctuated within 20% of the initial values and analyzed by pClamp 10.2 software (Molecular Devices) [67]. For the treatment of D1R agonist, SKF-38393 (10–50 μM) was applied for 5 min.

### Statistical analyses

All statistical analyses were performed with SPSS statistical software (version 20.0) or GraphPad Prism 7.00. Sample size was determined according to previously published studies [4, 8–10]. No animals were excluded. The normality of all data was analyzed using Shapiro-Wilk normality test. Levene’s test was used for the test of equal variances. For the data with normal distributions, two-tailed and unpaired Student’s *t* test was performed to analyze two independent groups with equal variance. Two-tailed and unpaired Student’s *t* test with Welch’s correction was used to analyze two independent groups with unequal variance. One-way ANOVA was performed to analyze multiple groups with only one variable and the differences between groups were performed with Tukey’s multiple comparisons test. For the data without normal distributions, Mann-Whitney *U* test was used to analyze two independent groups, Kruskal-Wallis test was used to analyze multiple groups and Dunn’s multiple comparisons test was used to analyze the differences between groups. Mixed design ANOVA with genotype as independent factor and stimuli/trials as repeated-measure factor was used to analyze different groups with two variables, including the time spent sniffing on different odors and latency to the platform. All results showed were mean ± SEM or median ± IQR, *n* represented the number of independent biological replicates and *p* value < 0.05 was considered significant. The statistical methods and statistical values of each result were presented in Additional file 3: Table S2.

## Supplementary information

**Additional file 1: Figure S1-9.** Figures and figure legends of supplementary figures.

**Additional file 2: Table S1.** List of primers used for qRT-PCR.

**Additional file 3: Table S2.** Statistical methods and values of all the results.

**Additional file 4: Table S3.** Data matrix for nontargeted metabolite analysis of PFC.

**Additional file 5: Table S4.** Alpha diversity and relative abundance values at various taxonomic levels of gut microbiota from 8-week-old WT and KO mice.

**Additional file 6: Table S5.** Alpha diversity and relative abundance values at various taxonomic levels of gut microbiota from 3/4-week-old WT and KO mice.

**Additional file 7: Table S6.** Alpha diversity and relative abundance values at various taxonomic levels of gut microbiota from 3-week-old C57BL/6J mice treated with fecal microbitoa from 8-week-old WT and KO mice.

**Additional file 8: Table S7.** Alpha diversity and relative abundance values at various taxonomic levels of gut microbiota from 3-week-old C57BL/6J mice treated with abx and fecal microbitoa from 8-week-old WT and KO mice.

**Additional file 9: Table S8.** Alpha diversity and relative abundance values at various taxonomic levels of gut microbiota from 6-week-old C57BL/6J mice treated with fecal microbitoa from 8-week-old WT and KO mice.

**Additional file 10: Table S9.** Alpha diversity and relative abundance values at various taxonomic levels of gut microbiota from 6-week-old C57BL/6J mice treated with abx.

**Additional file 11: Table S10.** Alpha diversity and relative abundance values at various taxonomic levels of gut microbiota from WT and KO mice treated with PBS or fecal microbitoa from WT mice.

**Additional file 12: Table S11.** The statistical values of histological changes of different tissues of WT and KO mice.

## Data Availability

The raw sequence data of 16S rRNA gene sequencing were deposited in the Sequence Read Archive (SRA) at NCBI under Bioproject PRJNA603256 (SRR10982902-SRR10982919, https://www.ncbi.nlm.nih.gov/bioproject/PRJNA603256). The data matrix for non-targeted metabolite analysis of PFC were supplied in Additional file 4: Table S3. All the other data in this work are available from the corresponding author upon reasonable request.

## Abbreviations

Abx: Pretreated with antibiotics (ampicillin, vancomycin, neomycin, metronidazole)
ACSF: Artificial cerebrospinal fluid
ASD: Autism spectrum disorder
Asp: Aspartic acid
DA: Dopamine
DOPAC: Dihydroxy-phenyl acetic acid
D1R: Dopamine D1 receptor
D2R: Dopamine D2 receptor
E/I imbalance: Excitation/inhibition imbalance
EP: Epinephrine
FITC: Fluorescein isothiocyanate
FMT: Fecal microbiota transplantation
GABA: Gamma-aminobutyric acid
GI: Gastrointestinal
Glu: Glutamic acid
Gly: Glycine
Gln: Glutamine
HPLC: High-performance liquid chromatography
5-HT: 5-Hydroxytryptamine
mPFC: Medial prefrontal cortex
NE: Norepinephrine
PLP: Pyridoxal 5'-phosphate
PM: Pyridoxamine
PN: Pyridoxine
VB6: Vitamin B_6_
VTA: Ventral tegmental area
Ser: Serine
sEPSCs: Spontaneous excitatory postsynaptic currents
sIPSCs: Spontaneous inhibitory postsynaptic currents
Th: Tyrosine hydroxylase
