# Supplementary material for: The gut microbiota regulates autism-like behavior by mediating vitamin B6 homeostasis in EphB6-deficient mice
Source: Microbiome. 2020 Aug 20;8:120. doi: 10.1186/s40168-020-00884-z (PMC7441571; doi:10.1186/s40168-020-00884-z)
Supplement: Supplementary file 2 — Additional file 1: Figure S1-9. Figures and figure legends of supplementary figures. [file 40168_2020_884_MOESM1_ESM.docx]

**
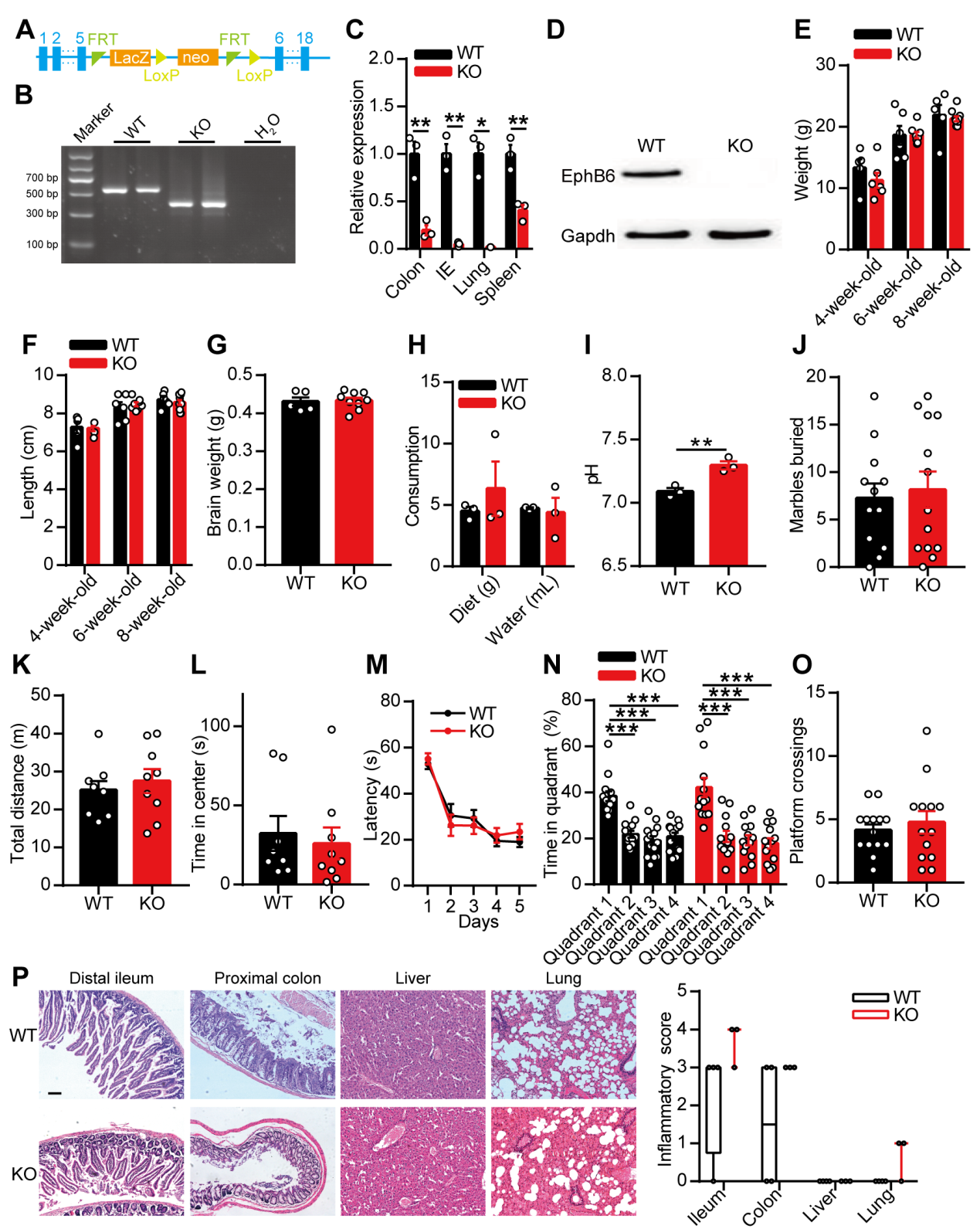
Figure S1 EphB6 knockout mice showed normal growth, motor behavior and spatial learning and memory**

(a) In EphB6 knockout mice, the inserted sequences were located in the intron between exon 5 and exon 6 of EphB6. (b) Data on genotyping of WT and KO mice using agarose gel electrophoresis were showed. The mutant band was 398 bp and the wild-type band was 542 bp. (c) The mRNA expression of EphB6 in different tissues of 8-week-old WT and KO mice was detected. n = 3 mice for each group. (d) The protein expression of EphB6 in brain of 8-week-old WT and KO mice was detected. (e-f) Similar body weight (e) and body length (f) between WT and KO mice were showed. n = 5-10 mice for each group. (g) Similar brain weight between WT and KO mice were showed. n = 5, 9 mice respectively. (h) Similar consumption of diet and water between WT and KO mice was showed. n = 3 mice for each group. (i) The fecal pH of 8-week-old WT and KO mice was detected. n = 3 mice for each group. (j) In marble burying test, 8-week-old WT and KO mice behaved similarly. n = 12, 13 mice respectively. (k-l) In open field test, total distances in 30 min (k) and time spent in center zone in 5 min (l) between 8-week-old WT and KO mice were similar. n = 8, 9 mice respectively. (m-o) In Morris water maze, 8-week-old KO mice spent same time finding the platform in acquisition test (m), spent same time in target quadrant (n) and had similar platform crossings (o) in probe test with WT mice. n = 14, 13 mice respectively. (p) The histological morphology of different organs of 8-weeks-old WT and KO mice by hematoxylin-eosin staining were showed. n = 4, 3 mice respectively. Scale bar was 50 μm. Data shown are mean ± SEM or median ± IQR. Two-tailed unpaired student’s *t* test (c, e-l, o), Mann Whitney test (p), one-way ANOVA (n), mixed design ANOVA with genotype as independent factor and stimuli/trials as repeated-measure factor (m). *, p < 0.05, **, p < 0.01, ***, p < 0.001. WT, EphB6^+/+^ mice; KO, EphB6^-/-^ mice, IE, intestinal epithelium. Statistical values are presented in Additional file 3: Table S2.

**
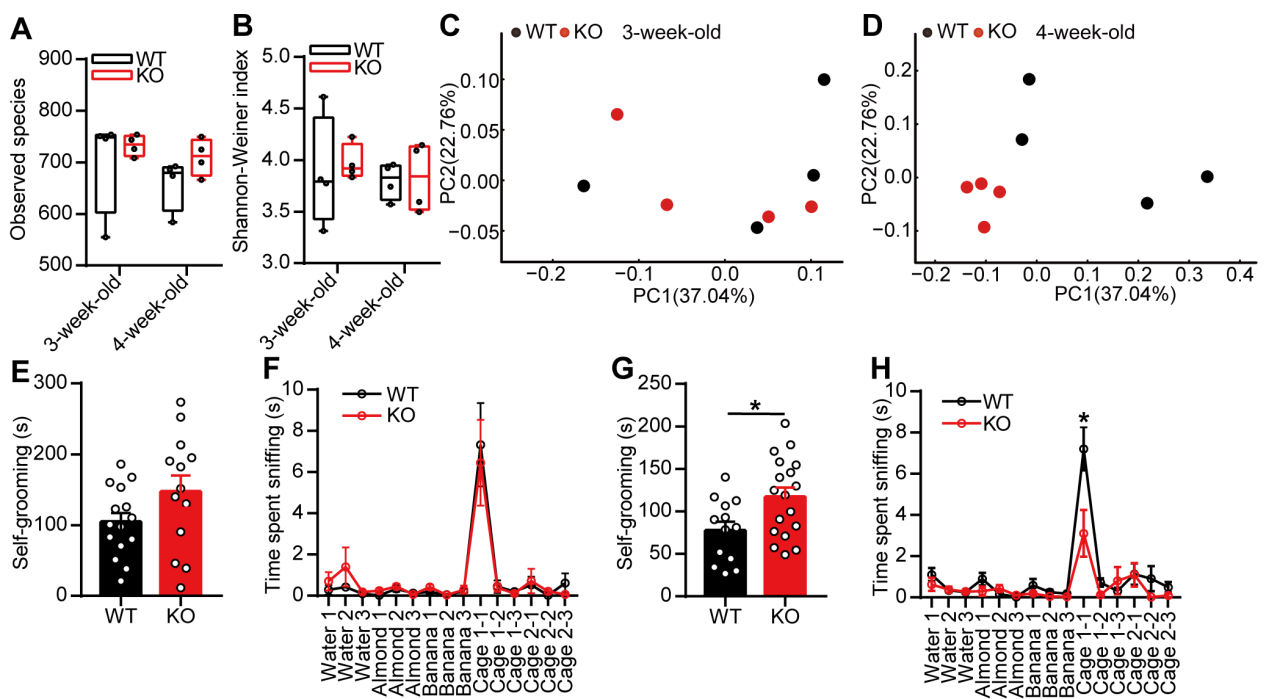
**

**Figure S2 4-week-old EphB6 ablation mice showed autism-like behavior and gut microbial disturbance**

(a-d) 16S rRNA gene sequencing of the gut microbiota from 3/4-week-old WT and KO mice. The species richness (a) and diversity (b) of the gut microbiota and the microbial composition (c-d) between the two groups were presented. n = 4 mice for each group. (e-f) Self-grooming test (e, n = 15, 13 mice respectively) and olfactory habituation/dishabituation test (f, n = 10, 6 mice respectively) in 3-week-old male WT and KO mice (21-23 days old) were conducted. (g-h) Self-grooming test (g, n = 13, 18 mice respectively) and olfactory habituation/dishabituation test (h, n = 10, 12 mice respectively) in 4-week-old male WT and KO mice (27-29 days old) were conducted. Data shown are mean ± SEM or medien ± IQR. Two-tailed unpaired student’s *t* test (e, g), Mann Whitney test (a-b), mixed design ANOVA with genotype as independent factor and stimuli/trials as repeated-measure factor (f, h), adonis analysis (c-d). *, p < 0.05. WT, EphB6^+/+^ mice; KO, EphB6^-/-^ mice. Statistical values are presented in Additional file 3: Table S2.


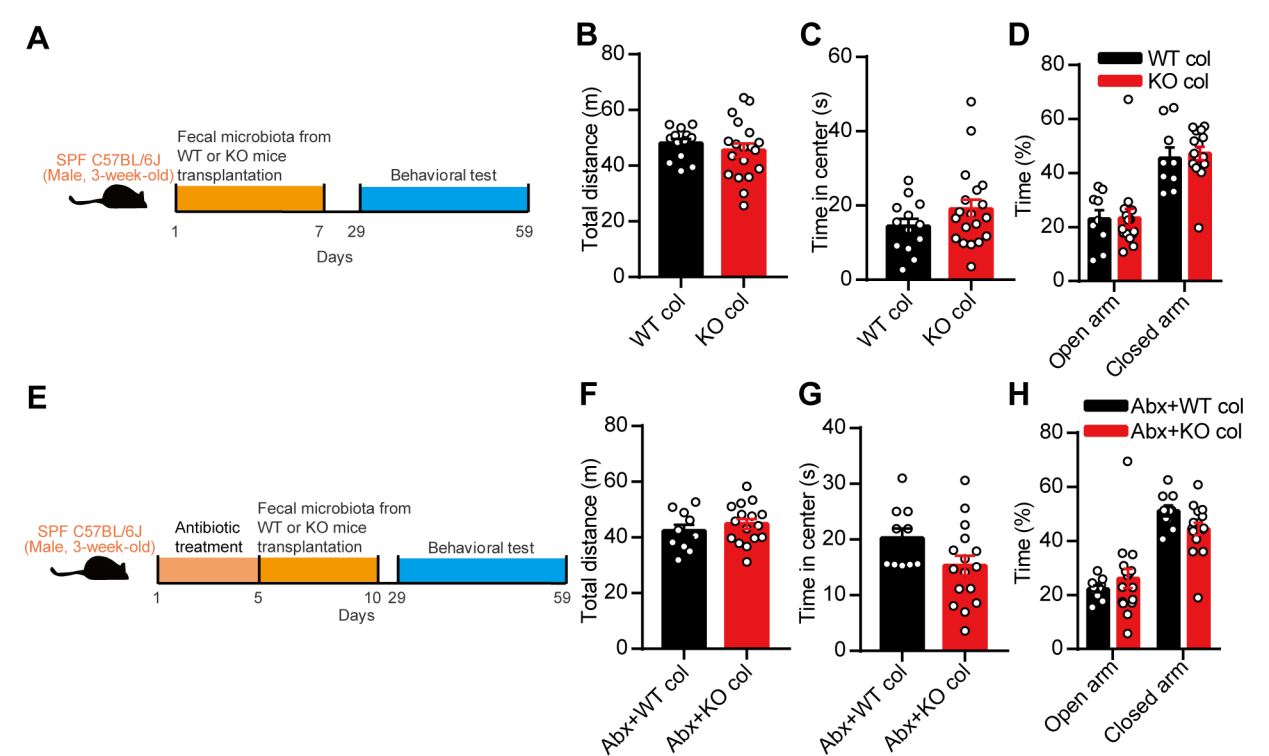


**Figure S3 C57BL/6J mice transplanted with the fecal microbiota from EphB6 ablation mice showed normal behavior in open field test and elevated plus maze**

(a-d) 3-week-old SPF male C57BL/6J mice were gavaged with the fecal microbiota from 8-week-old male WT and KO mice (a). Locomotor activities (b) and time in center zone (c) in open field test (b-c, n = 13, 19 mice respectively) were analyzed. Time spent in open arm and closed arm in elevated plus maze test (d, n = 9, 15 mice respectively ) was presented . (e-h) 3-week-old SPF male C57BL/6J mice were gavaged with antibiotics and the fecal microbiota from 8-week-old WT and KO mice (e). Locomotor activities (f) and time in center zone (g) in open field test, and time spent in open arm and closed arm in elevated plus maze test (h) were presented. n = 10, 16 mice respectively. Data shown are mean ± SEM. Two-tailed unpaired student’s *t* test (b-d, f-h). WT, EphB6^+/+^ mice; KO, EphB6^-/-^ mice; WT col or KO col, colonized with the fecal microbiota from EphB6^+/+^ mice or EphB6^-/-^ mice; Abx, pretreated with antibiotics (ampicillin, vancomycin, neomycin, metronidazole). Statistical values are presented in Additional file 3: Table S2.


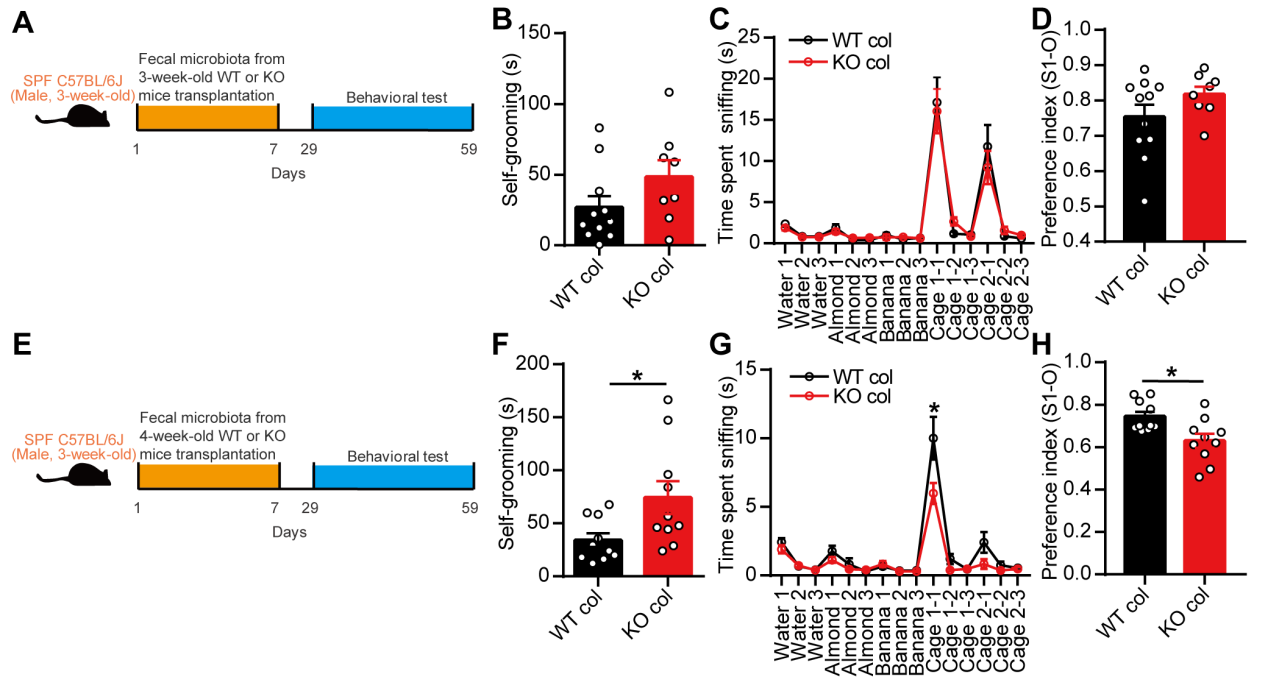


**Figure S4 Transplantation of the fecal microbiota from 4-week-old EphB6 ablation mice induced autism-like behavior in SPF C57BL/6J mice**

(a-d) The fecal microbiota from 3-week-old male WT and KO mice were gavaged to 3-week-old SPF male C57BL/6J mice for one week (a). After three weeks, self-grooming test (b), olfactory habituation/dishabituation test (c) and three-chambered social approach task (d) were conducted with an interval of at least 2 days. n = 11, 8 mice respectively. (e-h) The fecal microbiota from 4-week-old male WT and KO mice were gavaged to 3-week-old SPF male C57BL/6J mice for one week (e). After three weeks, self-grooming test (f), olfactory habituation/dishabituation test (g) and three-chambered social approach task (h) were conducted with an interval of at least 2 days. n = 10 mice for each group. Data shown are mean ± SEM. Two-tailed unpaired student’s *t* test (b, d, f, h), mixed design ANOVA with genotype as independent factor and stimuli/trials as repeated-measure factor (c, g). *, p < 0.05. WT, EphB6^+/+^ mice; KO, EphB6^-/-^ mice; WT col or KO col, colonized with the fecal microbiota from EphB6^+/+^ mice or EphB6^-/-^ mice. Statistical values are presented in Additional file 3: Table S2.

**
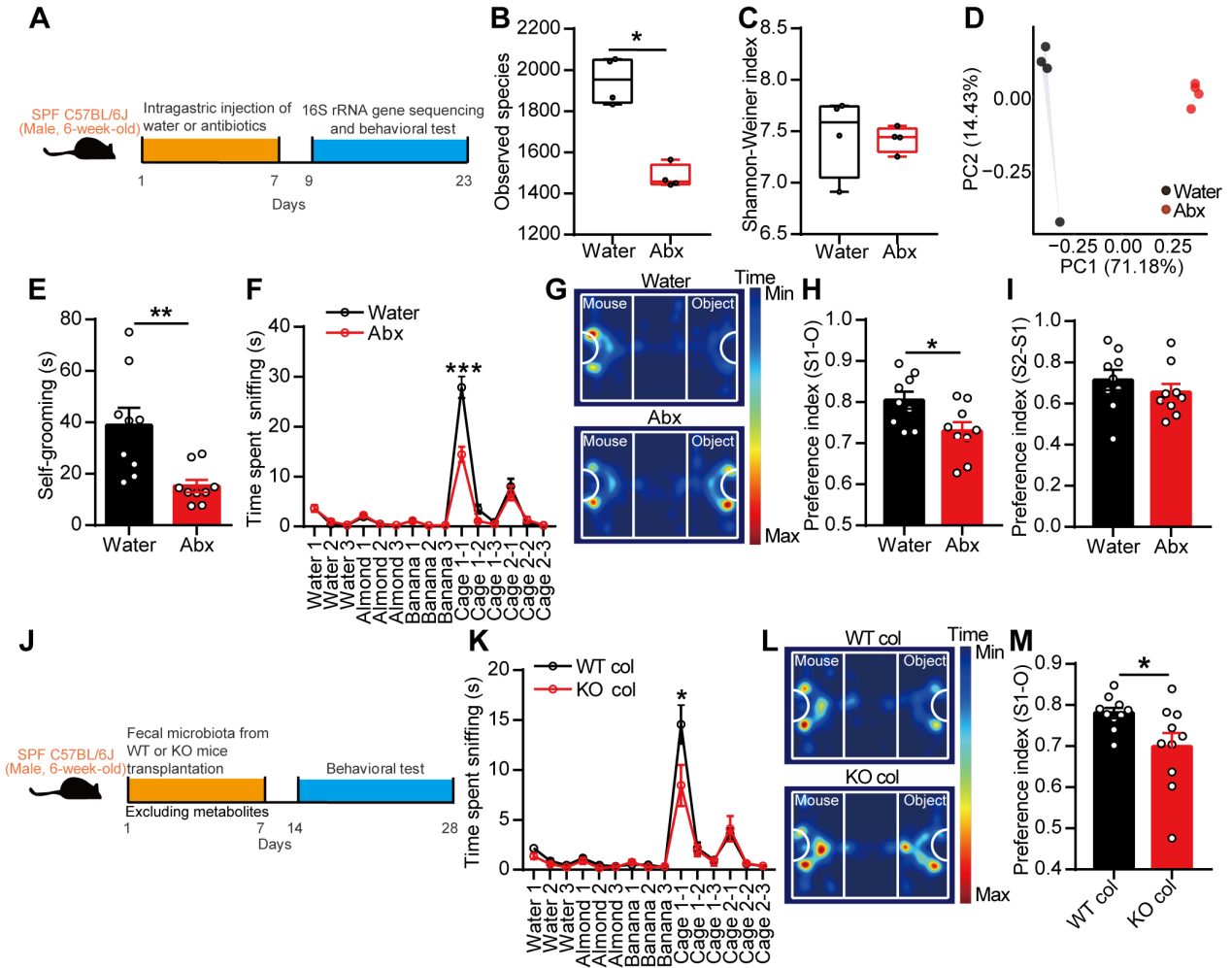
**

**Figure S5 Antibiotic treatment or gut microbiota without metabolites from EphB6 ablation mice induced partial social deficits in 6-week-old C57BL/6J mice**

(a-i) Schematic of the treatment of antibiotics (a). 6-week-old SPF male C57BL/6J mice were orally gavaged with antibiotics (ampicillin, vancomycin, neomycin, metronidazole) for 7 days. After 7 days, the fecal microbiota from the treated C57BL/6J mice were sequenced (b-d, n = 4 mice for each group) and self-grooming test (e), olfactory habituation/dishabituation test (f), three-chambered social approach task (g-i) were conducted with an interval of at least 2 days. n = 9 mice for each group. (j-m) The fecal microbiota from 8-week-old male WT and KO mice, in which metabolites were excluded, were orally gavaged to 6-week-old SPF male C57BL/6J mice for one week (j). After one week, olfactory habituation/dishabituation test (k) and three-chambered social approach task (l-m) were conducted with an interval of at least 2 days. n = 10 mice for each group. Data shown are mean ± SEM or medien ± IQR. Two-tailed unpaired student’s *t* test (e, h-i, m), Mann Whitney test (b-c), mixed design ANOVA with genotype as independent factor and stimuli/trials as repeated-measure factor (f, k), anosim analysis (d). *, p < 0.05; **, p < 0.01; ***, p < 0.001. Abx, treated with antibiotics (ampicillin, vancomycin, neomycin, metronidazole); WT col or KO col, colonized with the fecal microbiota from EphB6^+/+^ mice or EphB6^-/-^ mice. Statistical values are presented in Additional file 3: Table S2.

**
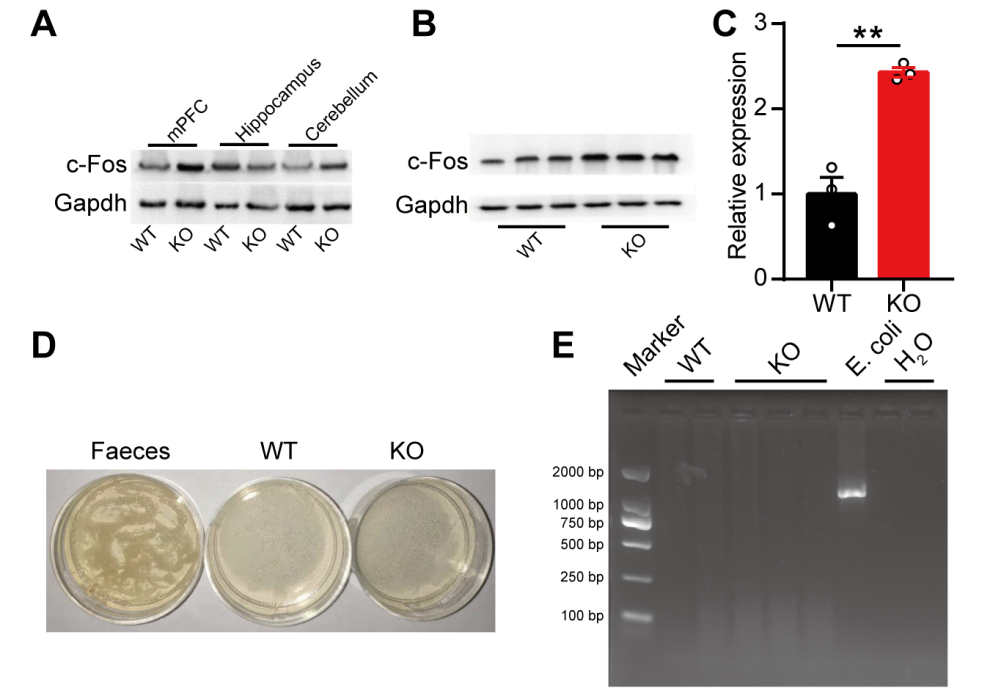
**

**Figure S6 The expression of c-Fos was increased in mPFC of KO mice performed with three-chambered social approach task**

(a-c) 1 hr after being performed three-chambered social approach task, the protein expression of c-Fos in mPFC (b-c, n = 3 mice for each group), hippocampus and cerebellum of WT and KO mice was detected. (d) Feces or PFC tissues of 8-week-old WT and KO mice were cultured in Luria-Bertani solid culture medium for 24 hr. n = 3,4 mice respectively. (e) DNA extracted from E. coli and PFC tissues of 8-week-old WT and KO mice were amplified by PCR using bacterial universal primers. n = 2, 3 mice respectively. Data shown are mean ± SEM. Two-tailed unpaired student’s *t* test (c). **, p < 0.01. WT, EphB6^+/+^ mice; KO, EphB6^-/-^ mice; mPFC, middle prefrontal cortex; PFC, prefrontal cortex; PCR, polymerase chain reaction; E. coli, *Escherichia coli*. Statistical values are presented in Additional file 3: Table S2.

**
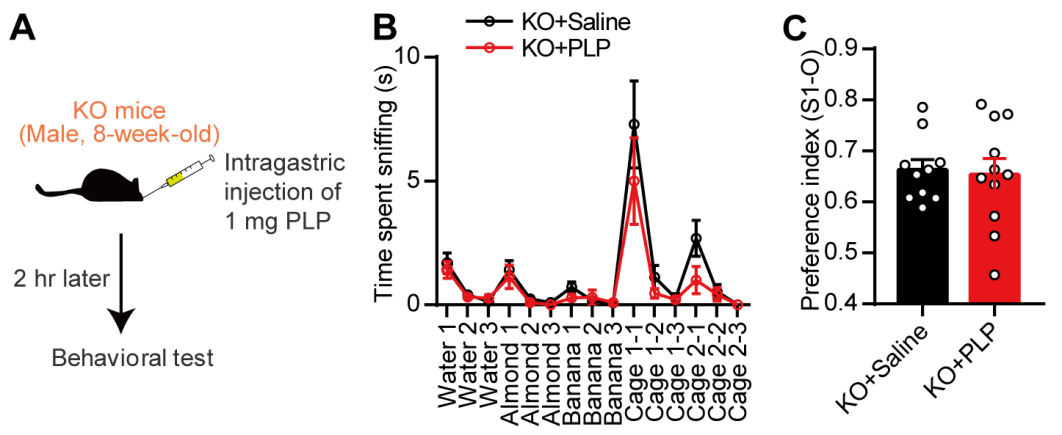
**

**Figure S7 Intragastric injection of vitamin B6 had no effect on social behavior in KO mice**

(a-c) 8-week-old male KO mice were gavaged with 1 mg PLP or saline. 2 hr later, olfactory habituation/dishabituation test (b, n = 12, 11 mice respectively) or three-chambered social approach task (c, n = 10, 11 mice respectively) was conducted. Data shown are mean ± SEM. Two-tailed unpaired student’s *t* test (c), mixed design ANOVA with genotype as independent factor and stimuli/trials as repeated-measure factor (b). KO, EphB6^-/-^ mice; PLP, pyridoxal 5’-phosphate. Statistical values are presented in Additional file 3: Table S2.

**
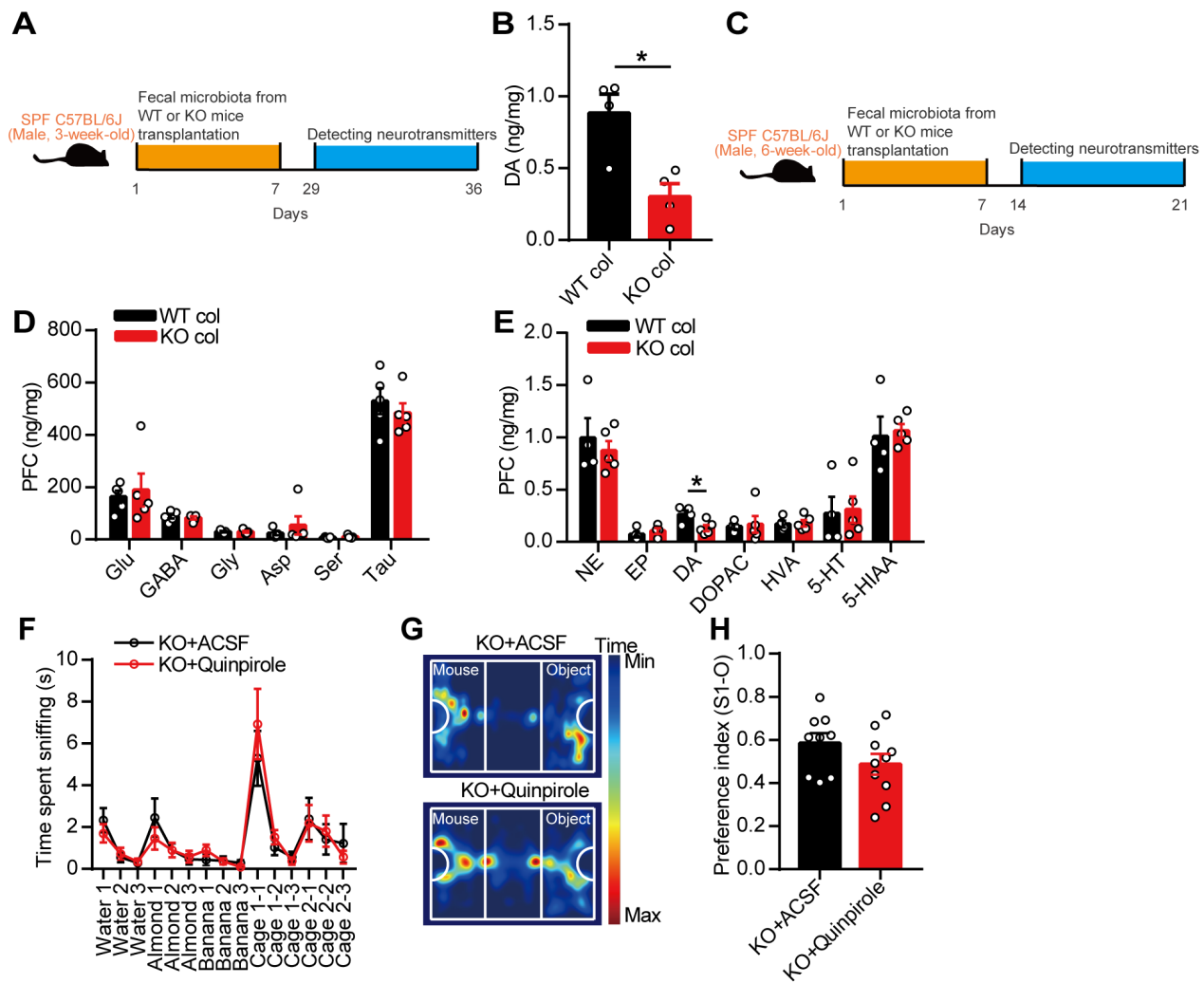
**

**Figure S8 Dopamine was mediated by gut microbiota in PFC of mice**

(a-b) The fecal microbiota from 8-week-old male WT and KO mice were gavaged to 3-week-old SPF male C57BL/6J mice for one week (a), then 3 weeks later, DA level in PFC of mice was detected (b). n = 4 mice for each group. (c-e) 6-week-old SPF male C57BL/6J mice were gavaged with the fecal microbiota from 8-week-old male WT or KO mice for one week (c), one week later, the amino acid neurotransmitters (d) and monoamine neurotransmitters (e) in PFC of mice were detected. n = 4-5 mice for each group. (f-h) 8-week-old male KO mice were injected with D2R agonist (quinpirole, 1 μg/0.3 μL) or ACSF in mPFC, then olfactory habituation/dishabituation test (f) and three-chambered social approach task (g-h) were conducted with an interval of one week. n = 9, 10 mice respectively. Data shown are mean ± SEM. Two-tailed unpaired student’s *t* test (b, d-e, h), mixed design ANOVA with genotype as independent factor and stimuli/trials as repeated-measure factor (f). *, p < 0.05. WT col or KO col, colonized with the fecal microbiota from EphB6^+/+^ or EphB6^-/-^ mice; PFC, prefrontal cortex; Glu, glutamic acid; GABA, gamma-aminobutyric acid; Gly, glycine; Asp, aspartic acid; Ser, serine; Tau, taurine; NE, norepinephrine; EP, epinephrine; DA, dopamine; 5-HT, 5-hydroxytryptamine; 5-HIAA, 5-hydroxyindoleacetic acid; DOPAC, dihydroxy-phenyl aceticacid; HVA, homovanillic acid; KO, EphB6^-/-^ mice; ACSF, artificial cerebrospinal fluid. Statistical values are presented in Additional file 3: Table S2.

**
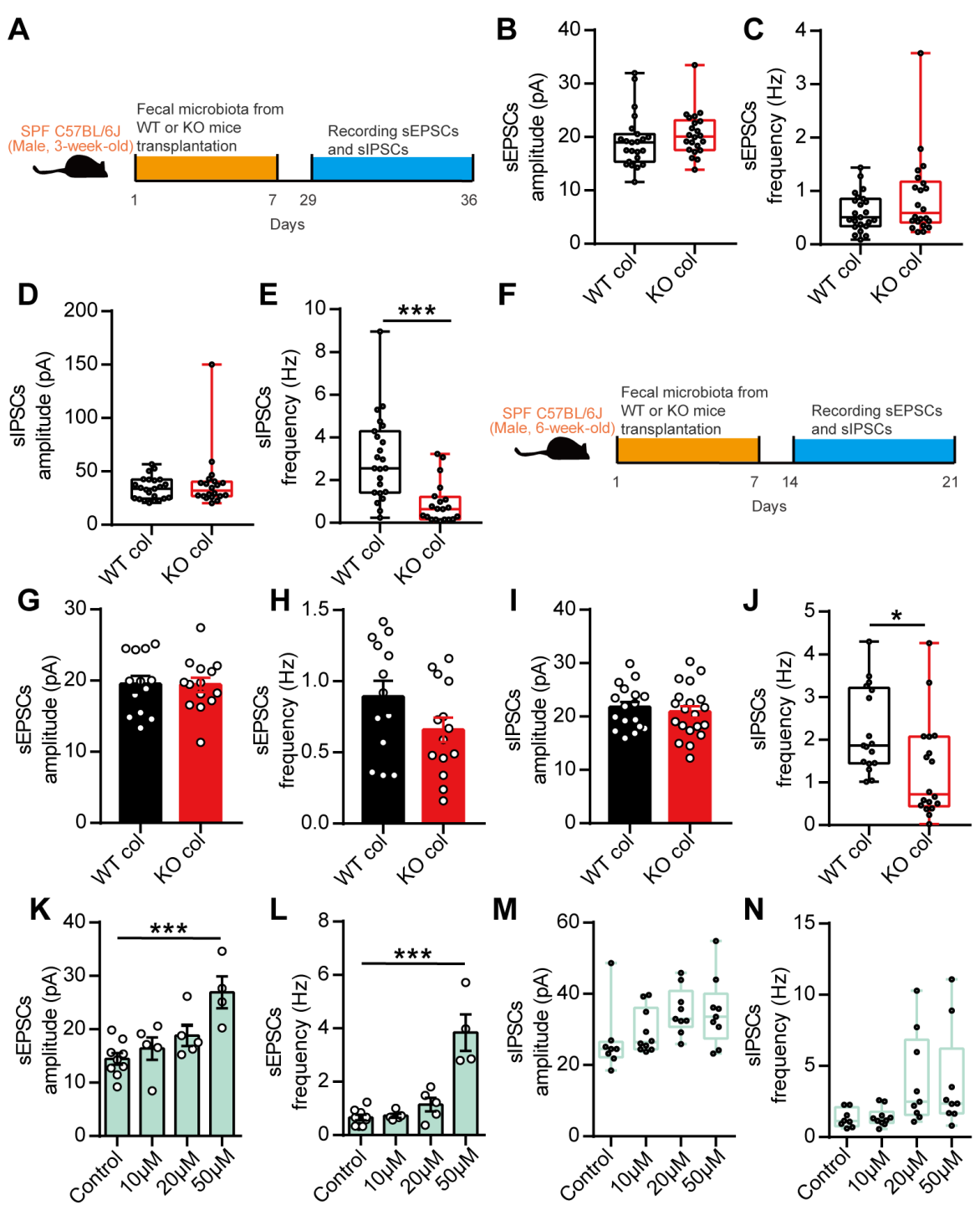
**

**Figure S9 The gut microbiota modulated E/I balance in mPFC of C57BL/6J mice**

(a-e) 3-week-old SPF C57BL/6J mice were gavaged with the fecal microbiota from 8-week-old WT and KO mice for one week (a), 3 weeks later, sEPSCs and sIPSCs of pyramidal neurons in mPFC of mice (b-e) were recorded. For sEPSCs, n = 23 and 22 cells from 4 mice respectively. For sIPSCs. n = 23 and 20 cells from 4 mice respectively. (f-j) 6-week-old SPF C57BL/6J mice were gavaged with the fecal microbiota from 8-week-old WT and KO mice for one week (f), one week later, sEPSCs and sIPSCs of pyramidal neurons in mPFC of mice (g-j) were recorded. For sEPSCs, n = 13 and 14 cells from 4 mice respectively. For sIPSCs. n = 17 and 18 cells from 4 mice respectively. (k-n) The mPFC slices were treated with different concentrations of D1R agonist, then sEPSCs and sIPSCs of pyramidal neurons in mPFC of 8-week-old WT mice were recorded. For sEPSCs, n = 9, 5, 5 and 4 cells from 4 mice respectively. For sIPSCs, n = 8, 10, 9 and 9 cells from 4 mice respectively. Data shown are mean ± SEM or median ± IQR. Two-tailed unpaired student’s *t* test (g-i), one-way ANOVA (k-l), Mann-Whitney test (b-e, j); Kruskal-Wallis test (m-n). *, p < 0.05; ***, p < 0.001. WT col or KO col, colocalized with the fecal microbiota from EphB6^+/+^ or EphB6^-/-^ mice; mPFC, middle prefrontal cortex; sEPSCs, spontaneous excitatory postsynaptic currents; sIPSCs, spontaneous inhibitory postsynaptic currents. Statistical values are presented in Additional file 3: Table S2.
